# High‐Voltage Cathode Materials for Sodium‐Ion Batteries: Advances and Challenges

**DOI:** 10.1002/smll.202501262

**Published:** 2025-04-17

**Authors:** Congqi Ren, Yulian Dong, Yong Lei

**Affiliations:** ^1^ Institute of Nanochemistry and Nanobiology School of Environmental and Chemical Engineering Shanghai University Shanghai 200444 China; ^2^ Fachgebiet Angewandte Nanophysik, Institut für Physik and IMN MacroNano Technische Universität Ilmenau 98693 Ilmenau Germany

**Keywords:** cathode materials, high‐voltage, polyanionic compounds, prussian blue analogs, sodium‐ion batteries, transition metal oxides

## Abstract

Sodium‐ion batteries (SIBs) gain attention as a promising, cost‐effective, and resource‐abundant alternative, especially for large‐scale energy storage. Cathode materials play a pivotal role in improving the electrochemical performance of SIBs, with high‐voltage cathodes providing enhanced energy density and rate capacity, making SIBs suitable for high‐power applications. Common cathode materials, such as layered transition metal oxides, polyanionic compounds, and Prussian blue analogs, each offer unique benefits. However, these materials face challenges under high‐voltage conditions, such as phase transitions, metal cation migration, oxygen loss, and electrolyte degradation. This review discusses strategies to address these challenges, including elemental doping, surface coatings, modified synthesis methods, and interfacial adjustments, all aimed at enhancing the stability and electrochemical performance of high‐voltage cathode materials. Here also explores how full‐cell design optimizations can further improve energy and power density. By analyzing material degradation and failure modes, this review offers insights into the development of stable, high‐performance SIBs with better safety and broader application potential in energy storage technologies.

## Graphical abstract

This review explores the role of high‐voltage cathode materials in enhancing the performance of SIBs. It focuses on strategies to improve energy density, rate capacity, and stability, addressing challenges like phase transitions, metal migration, and electrolyte degradation. The paper also highlights optimization techniques for full‐cell design and material durability, aiming for safer, high‐performance SIBs.

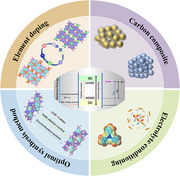

## 1 Introduction

The industry is dominated by lithium‐ion batteries (LIBs) because of their high energy density, extended cycle life, and exceptional rate performance, all of which are utilized on a daily basis.^[^ ^1^, ^2^ ^]^ However, the limited availability and uneven geographic distribution of lithium resources have led to rising costs, raising concerns about their long‐term sustainability and economic viability.^[^ ^3^, ^4^ ^]^ Sodium‐ion batteries (SIBs) are becoming more and more popular as an economical and resource‐rich substitute, especially for large‐scale energy storage applications.^[^ ^5^ ^]^ SIBs have benefits including better thermal stability and reduced cost. Research highlights that cathode materials play a critical role in improving the electrochemical performance of SIBs, with high‐voltage cathodes offering higher energy density and rate capacity, making SIBs suitable for high‐power applications.^[^ ^6^ ^]^

**Figure 1:**
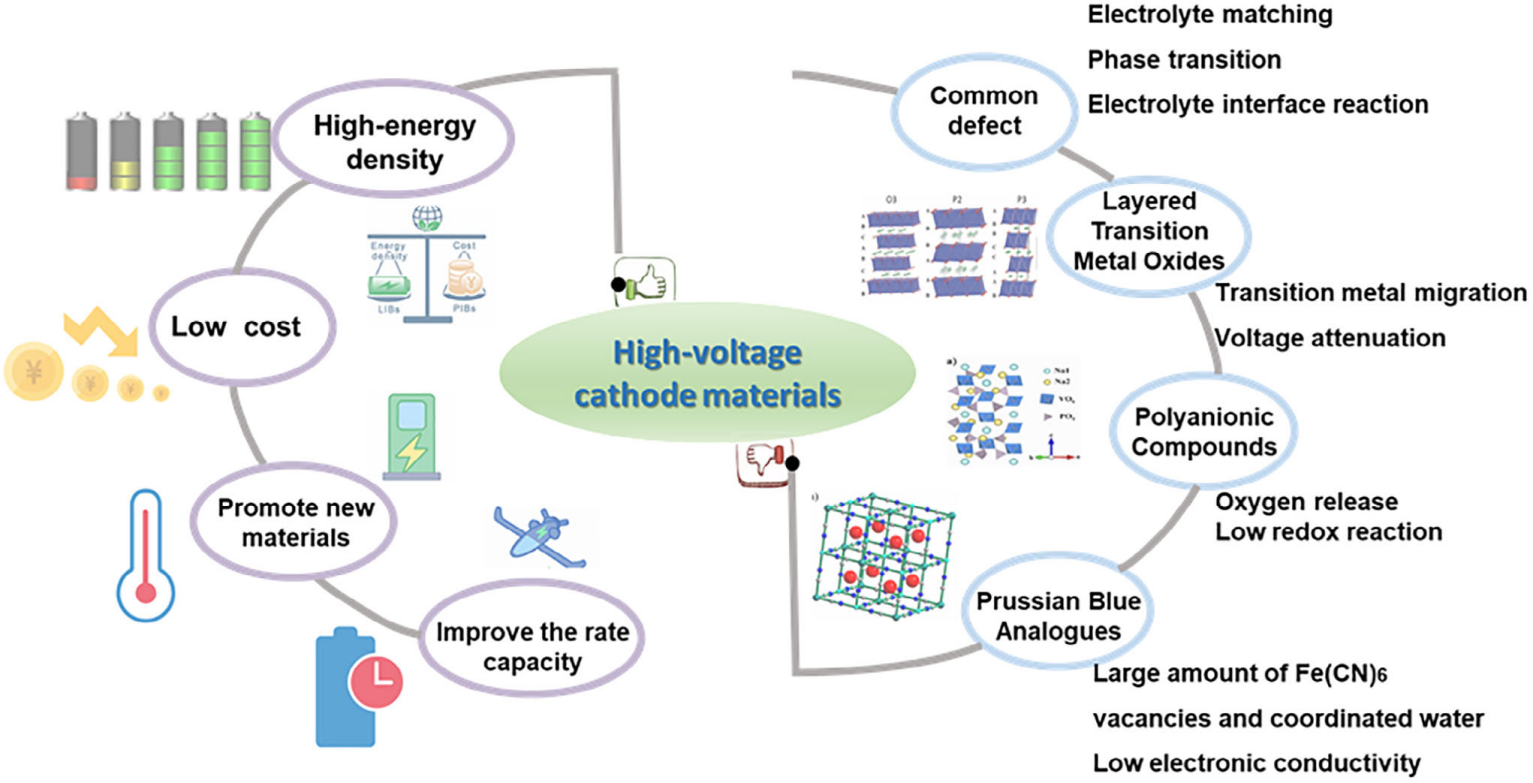
Advantages and disadvantages of high‐voltage cathode materials.

The primary materials under investigation include layered transition metal oxides, polyanionic compounds, and Prussian blue analogs (PBAs), each with distinct advantages, as shown in **Figure** **1**. Layered transition metal oxides exhibit excellent rate performance and cycling stability due to their large sodium layer spacing, which enhances ion transport.^[^ ^7^ ^]^ Polyanionic compounds offer robust structural frameworks, high thermal stability, and environmental resilience.^[^ ^8^ ^]^ PBAs are notable for their high sodium storage capacity, enabled by their open framework and large void spaces.^[^ ^9^ ^]^ Despite these advantages, cathode materials encounter significant challenges at high voltages. For example, the P2‐type structure of layered transition metal oxides can undergo a P2‐O2 phase transition at voltages above 4.2 V, causing lattice parameter changes that lead to particle rupture and performance degradation.^[^ ^10^ ^]^ Similarly, high‐valence TMs (e.g., Fe^4+^ and Ni^4+^) in polyanionic compounds may trigger redox reactions at elevated voltages,^[^ ^11^ ^]^ resulting in oxygen release, structural rearrangement, and capacity loss.^[^ ^12^ ^]^ In PBAs, vacancy defects in Fe(CN)_6_ units can cause structural collapse during charge–discharge cycles, reducing cycling stability.^[^ ^13^ ^]^ Additionally, high‐voltage conditions increase the risk of electrolyte decomposition, generating corrosive HF and accelerating structural degradation.^[^ ^14^ ^]^ Thus, stabilizing cathode materials and their interfaces under high‐voltage conditions is a key focus for advancing next‐generation battery technologies.

To enhance energy density while preserving high‐voltage stability, researchers have developed a range of functional strategies, including elemental doping, modified synthesis methods, surface coatings, and interfacial adjustments. These approaches aim to optimize the electrochemical properties and structural stability of cathode materials to increase the operating voltage. For instance, elemental doping can improve redox potential and theoretical energy density by introducing new active sites and stabilizing the crystal lattice.^[^ ^15^ ^]^ Modified synthesis methods, such as substituting phosphate with sulfate, have demonstrated increased redox potential and energy density.^[^ ^16^ ^]^ Surface coatings, on the other hand, play a crucial role in preventing electrolyte decomposition at high voltages.^[^ ^17^ ^]^ Coatings such as carbon, metal oxides, or fluorides form a protective layer on the cathode surface, mitigating side reactions and enhancing structural integrity during charge–discharge cycles.^[^ ^18^ ^]^ Similarly, interfacial adjustments involve engineering the electrode–electrolyte interface to improve ion transport and suppress parasitic reactions.^[^ ^19^ ^]^ These strategies may include tailoring the electrolyte composition, introducing cathode electrolyte interphases (CEIs), or modifying the cathode surface to promote compatibility with the electrolyte.^[^ ^20^, ^21^, ^22^ ^]^ By addressing challenges such as phase transition, capacity loss, structural instability, transition metal dissolution, and side reactions, these comprehensive modification strategies are pivotal for developing high‐voltage cathode materials.^[^ ^23^, ^24^ ^]^ Their implementation represents a significant step forward in advancing SIB technologies to meet the growing demands of energy storage applications.

Recent reviews have discussed cathode materials for SIBs, but most focus on specific material modifications and don't fully address their performance under high‐voltage conditions, which is key to advancing their development. High‐voltage cathode materials increase the voltage window by allowing higher charging and discharging voltages, boosting both operating voltage and energy density.^[^ ^25^ ^]^ Energy density is crucial for applications like electric automobiles and grid energy storage, as it determines battery range and efficiency.^[^ ^26^ ^]^ With an emphasis on layered transition metal oxides, polyanionic compounds, and PBAs, this review highlights the obstacles and advancements in high‐voltage cathode materials for SIBs with the goal of enhancing energy density and overall performance. We analyze key challenges that arise when the voltage limit increases, such as phase changes, metal cation migration, loss of reactive oxygen species, and electrolyte interaction. The review also examines methods to improve the stability of these materials under high voltage, including elemental doping, surface coatings, modified synthesis methods, and electrolyte optimization. Finally, we discuss how full‐cell design, such as electrode thickness and diaphragm material, can enhance energy and power density while ensuring practical reliability. In the last, by analyzing material degradation and failure modes, this review provides insights into designing stable, high‐performance SIBs with better safety and broader applications.

## 2 Types and Chemical Properties of High‐Voltage Cathode Material

### 2.1 Advantages of Cathode Materials at High Voltage

High‐voltage cathode materials offer several advantages, making them critical for advancing SIBs.

**Figure 2:**
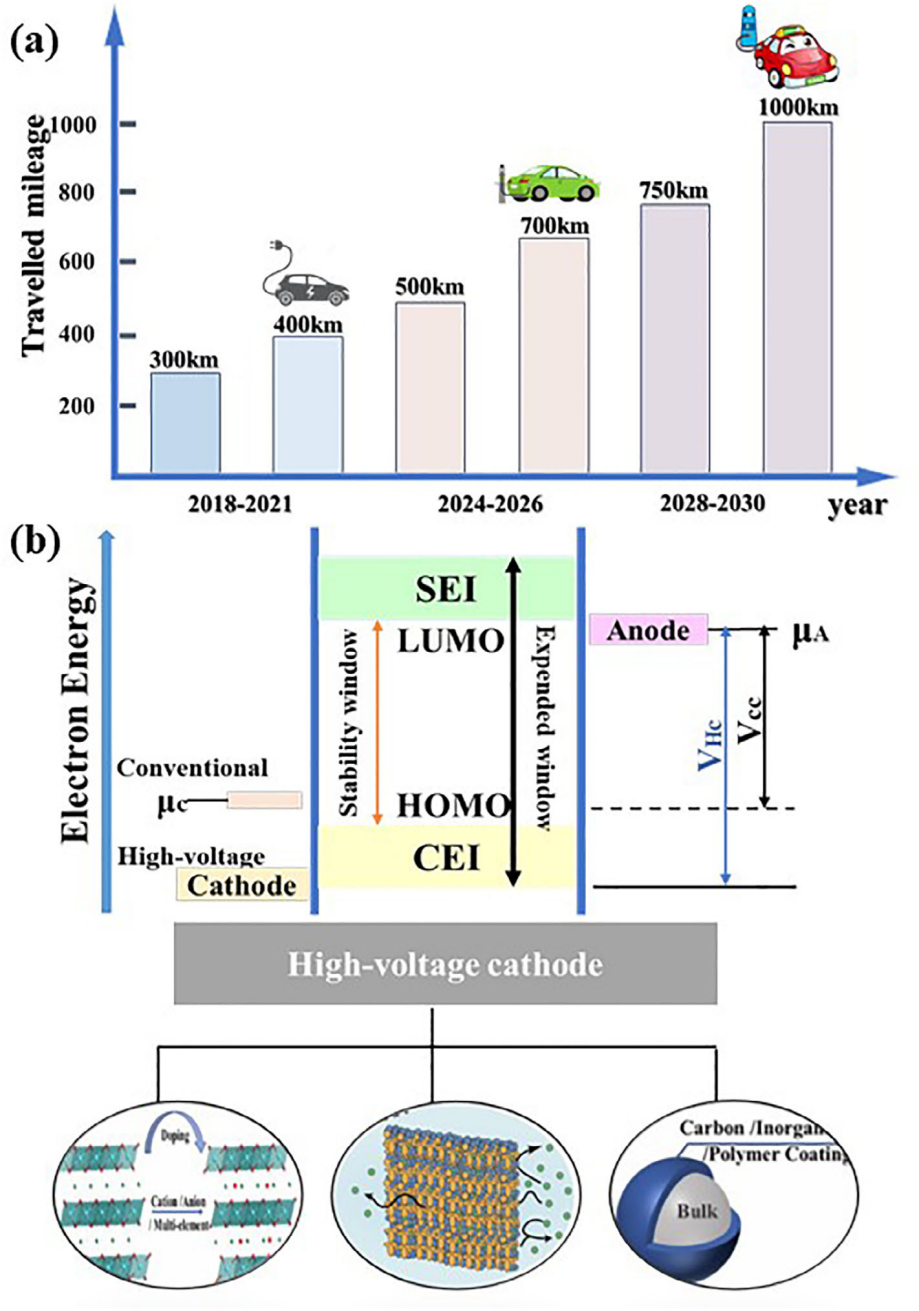
a) Enhanced energy density on the increased range of electric vehicles over recent years. b) Effects of design modifications on cathode voltage improvement and development of new materials under high‐voltage platforms.

i) Enhancement of energy density: High voltage significantly improves energy density, calculated as the product of operating voltage and specific capacity.^[^ ^27^ ^]^ The operating voltage, *E*, is expressed as:
(1) $$E=E_{Cathode}-E_{Anode}=\frac{\Delta G}{\mathrm{nF}}$$

where *E*_Cathode_ and *E*_Anode_ are the cathode and voltages, *ΔG* is the Gibbs free energy difference, 𝑛 is the electron transfer number, and 𝐹 is the Faraday constant. The theoretical specific capacity, *Q*, can be calculated as:

(2) $$Q=\frac{nF}{3.6Mw}$$

where *M_w_* is the molar mass. Increasing the electron transfer number or decreasing the molecular weight are two methods to raise the energy density. High‐voltage platforms also improve power density, which is essential for applications.^[^ ^28^ ^]^ Increased energy density in electric vehicles can provide the advantage of increased range, reduced bulk weight, and improved charging efficiency (**Figure** **2**a).^[^ ^29^ ^]^

ii) Cost reduction: High‐voltage cathode materials reduce battery costs by enabling the substitution of expensive elements like vanadium with more affordable options such as iron. Increased energy density also minimizes the material required for equivalent energy storage, further decreasing cost and weight.^[^ ^30^, ^31^ ^]^
iii) Promote the development of new materials: High‐voltage cathodes encourage the development of novel materials and improve the adaptability of existing ones. Modifications enhance energy density and cycle stability under high‐voltage conditions while improving performance at extreme temperatures.^[^ ^32^ ^]^ These strategies simultaneously advance cathode voltage and foster new material innovation (Figure 2b).
iv) Improvement of electrochemical performance: Elemental doping (e.g., Mg^2+^,^[^ ^33^ ^]^ K^+^,^[^ ^34^ ^]^ F^−^,^[^ ^35^ ^]^) strengthens ionic bonds, reduces oxygen loss, and enhances structural stability during charge–discharge cycles, improving cycling performance under high‐voltage conditions.^[^ ^36^ ^]^
v) Better rate performance and cycle life: Optimizing structure and interfaces in high‐voltage cathodes enhances rate performance and extends cycle life.^[^ ^37^ ^]^ For example, Na_4_Co_3_(PO_4_)_2_P_2_O_7_/CNT composites modified by Al^3+^ doping and carbon nanotube integration exhibit superior high‐rate performance.^[^ ^38^ ^]^

High‐voltage cathode materials are pivotal for advancing SIBs due to their ability to significantly enhance energy density, reduce costs, and promote the development of innovative materials. These materials meet the increasing needs of applications like grid energy storage and electric cars by maximizing electrochemical performance, structural stability, and energy economy.

### 2.2 Challenges for Cathode Materials at High Voltage

High‐voltage cathode materials offer numerous advantages, yet they face persistent challenges related to stability, safety, cycle life, and cost. Among the three primary types of cathode materials, layered transition metal oxides, polyanionic compounds, and PBAs, common issues arise that hinder their practical application. Safety remains a significant concern, as external factors such as overcharging or overheating can initiate self‐heating processes within batteries.^[^ ^39^ ^]^ These processes trigger a cascade of chemical reactions, including electrolyte decomposition and interactions with electrode materials, leading to surface reconstruction, capacity degradation, and excessive internal voltage.^[^ ^40^ ^]^ These reactions create serious safety issues by increasing the possibility of thermal runaway and, in the worst situations, explosions.^[^ ^41^ ^]^

Interface stability also plays a pivotal role in the electrochemical performance of high‐voltage cathodes. Hydrogen transfer between anions (e.g., PF_6_^−^, BF_4_^−^, and ClO_4_^−^) and solvents generates acids, such as HF and HClO_4_, which corrode transition metal ions, forming transition metal fluorides (TMF*_x_*).^[^ ^42^ ^]^ This corrosion destabilizes the CEI, leading to the dissolution of TMs into the electrolyte, accelerating structural degradation and capacity decay.^[^ ^43^ ^]^ Moreover, the inhomogeneous formation of CEI exacerbates issues by creating uneven current densities, triggering localized overheating and stress concentrations, particularly at elevated temperatures. These conditions further compromise safety and contribute to battery degradation.^[^ ^44^ ^]^

Another critical challenge is the compatibility of high‐voltage cathode materials with existing electrolytes. Oxidative decomposition and limited electrolyte stability often result from operating potentials that exceed the stabilization window of carbonate‐based electrolytes.^[^ ^45^ ^]^ This mismatch leads to low Coulombic efficiency, poor cycling stability, and diminished reversible capacity. Addressing these issues requires the development of advanced electrolytes with a broader electrochemical stabilization window and the incorporation of suitable additives to enhance performance.^[^ ^46^ ^]^ Overcoming these obstacles is essential for the large‐scale commercialization of high‐voltage cathode materials and their successful integration into SIBs.

#### 2.2.1 Challenge of Layered Transition Metal Oxides

Layered transition metal oxides face considerable challenges under high‐voltage conditions, particularly concerning transition metal migration and voltage decay. At elevated voltages, the decomposition of electrolytes generates HF, which facilitates the dissolution of transition metal ions, especially Ni and Mn, due to the Jahn–Teller effect.^[^ ^47^ ^]^ These dissolved ions migrate into the electrolyte, leading to adverse effects such as irreversible anionic redox reactions, voltage hysteresis, and poor cycling stability.^[^ ^48^ ^]^ The migration of transition metals undermines the electrochemical performance and structural integrity of the material, posing a significant hurdle for achieving stable, high‐voltage operation.^[^ ^49^ ^]^

Voltage decay is another critical issue for layered oxides, arising from the interplay between oxygen release and changes in the valence states of transition metals. Differences in the energy levels of redox pairs strongly influence the extent of voltage decay.^[^ ^50^ ^]^ For instance, the reductions of Mn and Co contribute prominently due to their large energy disparities. Oxygen release exacerbates this decline by activating redox pairs such as Mn^3+^/Mn^4+^ and Co^2+^/Co^3+^ for charge compensation, which induces structural damage. The release of oxygen also alters particle morphology, causing fractures and increasing exposed surface areas, further degrading the cycling stability and lifespan of these materials.^[^ ^51^ ^]^

#### 2.2.2 Challenges of Polyanionic Compounds

Polyanionic compounds exhibit distinct challenges under high‐voltage conditions, primarily due to slow redox reaction kinetics and oxygen release. The rigid structure of polyanionic cathodes often hinders redox reaction kinetics, reducing charge transfer rates during charging and discharging cycles.^[^ ^52^ ^]^ This sluggish behavior limits the energy efficiency and applicability of these materials in high‐performance systems.

Additionally, at elevated voltages, high‐valence transition metals such as Fe^4+^ and Ni^4+^ can participate in redox reactions that release oxygen. This oxygen release leads to structural rearrangements and irreversible capacity loss, further impairing the electrochemical performance of polyanionic compounds. The simultaneous occurrence of electrolyte oxidation compounds these issues, accelerating degradation and reducing the material's overall stability.^[^ ^47^ ^]^

#### 2.2.3 Challenges of Prussian Blue Analogs

PBAs confront a unique set of challenges under high voltage, including structural defects, slow redox kinetics, and poor electronic conductivity. During synthesis, Fe(CN)_6_ vacancy defects and crystalline water are commonly introduced, which compromise material performance.^[^ ^53^ ^]^ Crystalline water occupies sodium storage sites and blocks ion transport channels, while Fe(CN)_6_ defects weaken structural stability, especially during charge–discharge cycling.^[^ ^54^ ^]^ At high voltages, these structural imperfections exacerbate electrolyte decomposition, HF release, and subsequent material degradation, significantly reducing battery efficiency.

The redox reaction kinetics in PBAs are also hindered under high voltage, leading to low charge–discharge efficiency. Transition metal ions such as Mn^3+^ are particularly susceptible to Jahn–Teller distortion, which worsens the cycling performance of the material.^[^ ^55^ ^]^ Furthermore, the absence of strong bonds between metal ions and ligands causes PBAs to have low electrical conductivity.^[^ ^56^ ^]^ High voltage aggravates this problem by inducing structural collapse, further disrupting sodium storage and electronic pathways. These combined effects accelerate the decline in electrochemical performance, highlighting the need for innovative approaches to address these limitations.^[^ ^57^ ^]^

Although PBAs, polyanionic compounds, and layered transition metal oxides have the potential as high‐voltage cathode materials for SIBs, each class has unique difficulties. To solve these issues, a deep comprehension of the structural, electrochemical, and interfacial processes that occur at high voltages is required. Future advancements in material design, synthesis techniques, and electrolyte optimization are crucial to overcoming these challenges and achieving the full potential of high‐voltage cathode materials.

### 2.3 Strategies for Overcoming Challenges at High Voltage

**Figure 3:**
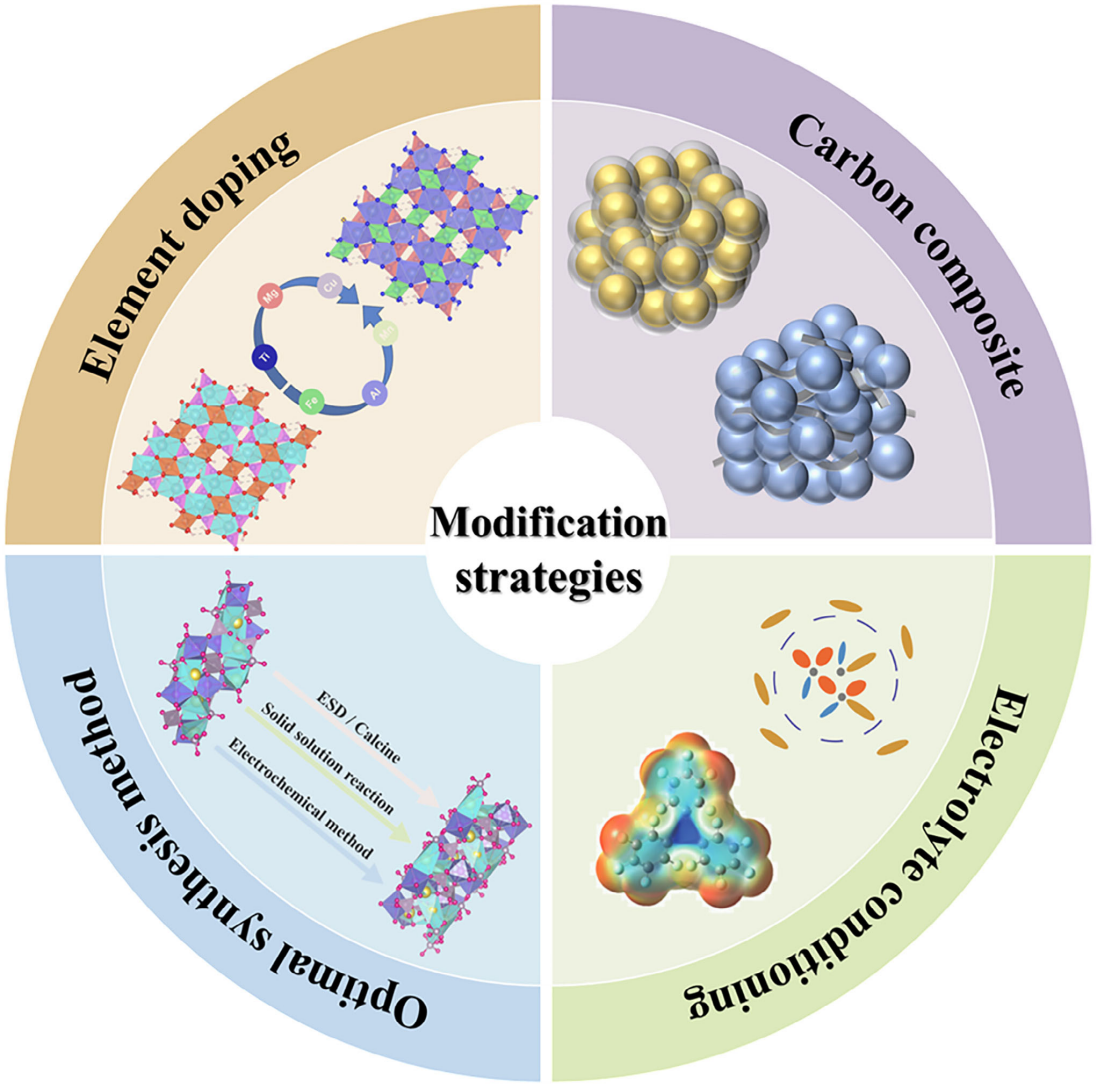
Strategies to enhance the performance and stability of high‐voltage cathode materials.

To address the challenges associated with high‐voltage cathode materials for SIBs, several design strategies have been proposed, including elemental doping, surface coating, electrolyte optimization, and improved synthesis methods (**Figure** **3**). These strategies target various performance parameters, such as structural stability, electronic conductivity, ion transport efficiency, and resistance to degradation under high voltage. By leveraging these approaches, the operational voltage, cycling stability, and overall electrochemical performance of cathode materials can be significantly enhanced.

#### 2.3.1 Elemental Doping

Elemental doping, including both cationic and anionic doping, plays a pivotal role in improving the redox activity and structural stability of high‐voltage cathode materials. Cation doping introduces elements with higher redox potentials (e.g., Li^+^, Mg^2+^, and K^+^), which enhances charge transfer at higher voltages and helps stabilize the material under the stress of high voltage cycling.^[^ ^58^ ^]^ This doping can mitigate volumetric changes (expansion or contraction) by providing structural backbones that prevent the material from collapsing. Additionally, doping can regulate the physicochemical properties of the crystal, facilitating efficient sodium‐ion transport while maintaining structural integrity, thus reducing voltage hysteresis and enhancing cycling performance.^[^ ^59^ ^]^

Anion doping, on the other hand, adjusts the electronic structure of the cathode material, boosting its operating voltage by modifying the energy band and Fermi energy levels.^[^ ^60^ ^]^ Electronegative elements like fluorine (F) enhance structural stability by forming strong bonds with metal ions, which prevents undesirable side reactions at the electrode/electrolyte interface.^[^ ^61^ ^]^ This doping increases the redox potential of certain redox pairs, further improving the electrochemical performance. The synergy between cationic and anionic doping allows for both structural and electrochemical enhancements, making doping an essential strategy for advancing high‐voltage cathode materials.

#### 2.3.2 Surface Coating

Surface coating, particularly with carbon composites (e.g., graphite, graphene oxide, and carbon nanotubes), complements doping strategies by improving conductivity and mitigating mechanical strain. These coatings form conductive networks that enhance electron diffusion and Na^+^ transport, preventing particle aggregation and promoting faster charge transfer.^[^ ^62^ ^]^ Carbon materials also act as elastic cushions, alleviating the stress induced by volume changes during cycling. This is critical for maintaining stable performance at high voltages.^[^ ^63^ ^]^

When combined with elemental doping, surface coatings provide a comprehensive solution that enhances both the bulk and surface properties of the cathode materials. The conductive framework provided by carbon coatings complements the structural enhancements from doping, ensuring the material can handle high voltages while maintaining stability and performance over extended cycles.

#### 2.3.3 Electrolyte Optimization

Electrolyte optimization is another crucial strategy for high‐voltage SIBs. By using electrolytes with higher oxidative stability, the electrochemical window is expanded, allowing the cathode materials to operate at higher voltages without experiencing electrolyte decomposition.^[^ ^64^ ^]^ This prevents side reactions at the electrode/electrolyte interface, leading to longer battery life and enhanced stability.^[^ ^65^ ^]^

The incorporation of specific additives, such as antioxidant solvents, also protects the cathode material from degradation by forming stable CEIs.^[^ ^66^ ^]^ These additives prevent the dissolution of transition metals and the loss of lattice oxygen, thereby improving the material's cycling performance.^[^ ^67^, ^68^ ^]^ Furthermore, solvation strategies, including the use of weakly solvated electrolytes (WSEs), regulate the solvation structure of sodium ions, improving the ionic conductivity and extending the high‐voltage stability of the electrolyte.^[^ ^69^ ^]^

#### 2.3.4 Optimization of Synthesis Methods: Tailoring Material Properties

The synthesis method has a significant influence on the structure and performance of cathode materials. By controlling the morphology, particle size, crystal structure, and crystallinity, synthesis methods directly impact the material's voltage stability, ion diffusion kinetics, and energy density.^[^ ^70^ ^]^ For example, advanced synthesis techniques like electrostatic spray deposition (ESD) enable the fabrication of self‐supported 3D porous structures that enhance redox activity, providing higher voltage and improved cycling stability.^[^ ^71^ ^]^

The morphology of the material can also influence the effectiveness of doping and coating strategies. Well‐ordered and crystalline structures ensure better ion diffusion and more stable electrochemical performance. When combined with optimized doping and coating strategies, these materials exhibit superior electrochemical behavior under high voltage conditions.^[^ ^72^ ^]^

The combination of doping, surface coating, electrolyte optimization, and advanced synthesis methods forms a cohesive strategy for improving the performance of high‐voltage cathode materials. These strategies complement and enhance each other, addressing different challenges related to structural stability, electrochemical activity, and interface integrity. By carefully tuning these factors, high‐voltage cathodes can achieve greater operating voltages, improved stability, and extended cycling life, thereby making SIBs more competitive for next‐generation energy storage applications.

## 3 Design Principles and Synthesis Strategies for High‐Voltage Cathode Materials

### 3.1 Structure Optimization and Doping Modification

#### 3.1.1 Cationic Doping

Cation doping replaces sodium ions or transition metal (TM) ions in a crystal with other metal cations, such as Mn, Fe, Ti, Cu, Al, or Zr. This process expands the crystal lattice and adjusts the crystal's intrinsic physicochemical properties. These modifications enable efficient Na^+^ transport while maintaining structural integrity, thereby reducing voltage hysteresis and enhancing cycling performance during charge–discharge cycles. Cation doping strategies are classified as low, medium, or high entropy based on the number and proportions of dopant elements.^[^ ^73^ ^]^ High‐entropy materials contain at least five primary elements with similar atomic proportions, medium‐entropy materials have three or four elements, and low‐entropy materials involve two or fewer.^[^ ^59^ ^]^

High‐entropy alloys offer remarkable properties, including superior mechanical characteristics and thermodynamic benefits, compared to conventional materials.^[^ ^74^ ^]^

The high‐entropy strategy enhances structural stability by reducing Gibbs free energy and modifying the crystal structure through the introduction of diverse elements into the lattice, thereby increasing conformational entropy.^[^ ^75^ ^]^ This approach integrates the benefits of entropic effects and doping, effectively preventing harmful phase transitions. As a result, cathode materials exhibit improved structural stability, ion/electron transport, prolonged cycle life, and faster charge/discharge rates.^[^ ^76^ ^]^

**Figure 4:**
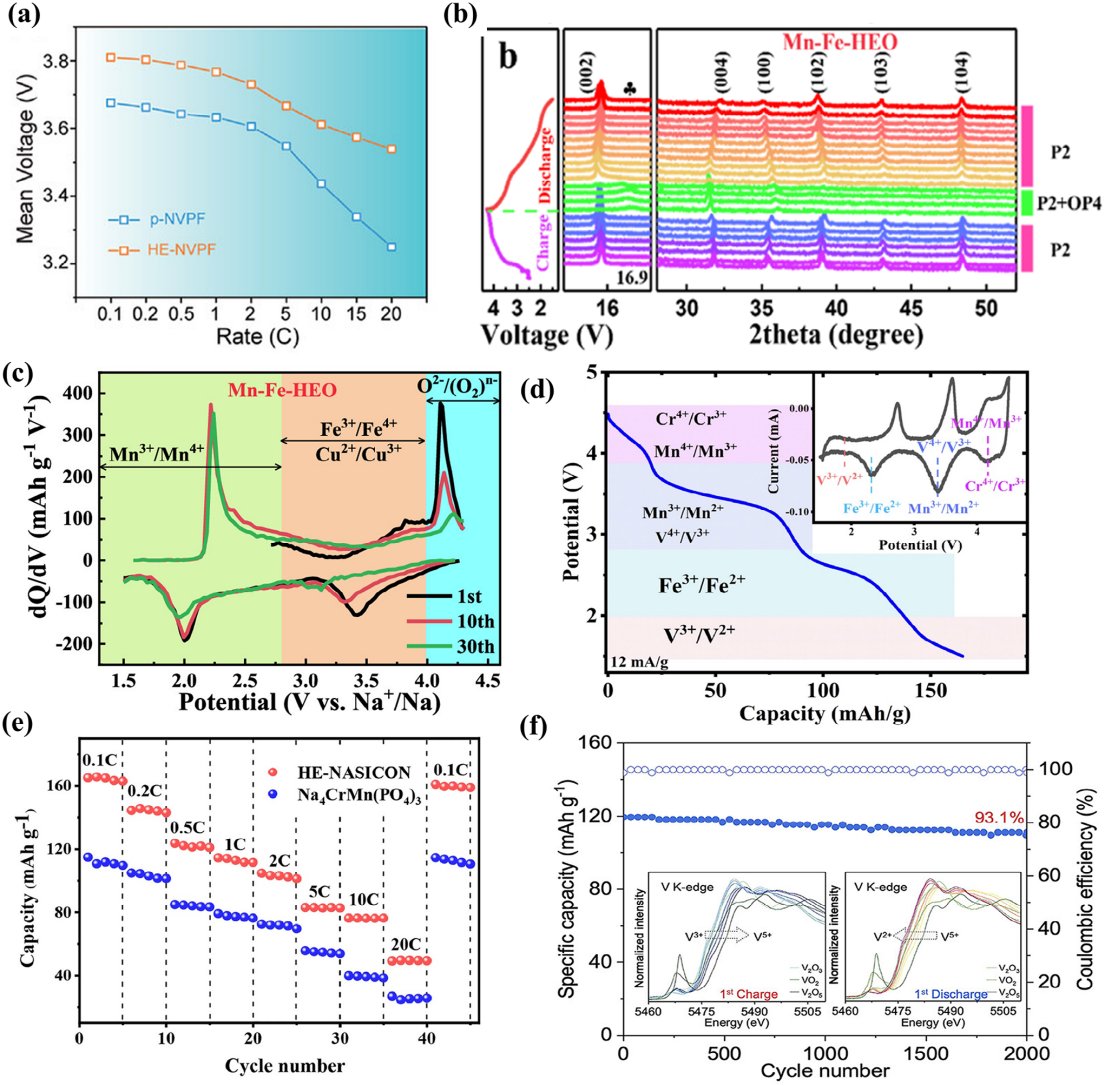
a) The voltage of HE‐NVPF and p‐NVPF at different rates. Reproduced with permission.^[^ ^77^ ^]^ Copyright 2022 Wiley‐VCH GmbH. b) Charge/discharge profiles and dQ/dV curves of Mn‐Fe‐HEO samples at 0.1 C at different cycle numbers. c) Ex situ XRD patterns for Mn‐Fe‐HEO samples during the first charge/discharge process. Reproduced with permission.^[^ ^78^ ^]^ Copyright 2024 American Chemical Society. d) Galvanostatic discharge profiles at a current density of 0.1 C of HE‐NASICON. e) Rate the performance of the HE‐NASICON from 0.1 to 20 C. Reproduced with permission.^[^ ^79^ ^]^ Copyright 2024 Elsevier B.V. f) Charge and discharge process of typical V K‐edge XANES spectra and cycling performance of the HE‐V1.6∥NG full cell at 2 A g^−1^. Reproduced with permission.^[^ ^80^ ^]^ Copyright 2024 American Chemical Society.

Researchers have incorporated the high‐entropy concept into cathode materials to improve electrochemical performance. Gu et al. adopted a high‐entropy substitution approach to develop a high‐entropy phosphoric acid cathode, Na_3_V_1.9_ (Ca, Mg, Al, Cr, Mn)_0.1_(PO_4_)_2_F_3_ (HE‐NVPF).^[^ ^77^ ^]^ This technique modified the crystal framework of NVPF while keeping the core active V atoms unchanged. The high‐entropy effect facilitated disordered Na^+^ rearrangement in the Na(2) site, mitigating discharge issues in low‐voltage regions. As illustrated in **Figure** **4**a, the voltage plateau exhibited a significant increase, shifting from 3.67 to 3.81 V. This enhancement contributed to greater Na storage capacity and higher energy density, which improved from 395.78 to 445.42 Wh kg^−1^. The cell preserved 80.4% of its capacity after 2000 cycles at 20 C and retained 60% at 50 C. When paired with an HC anode, the HE‐NVPF//HC sodium‐ion full cell attained a specific energy density of 326.8 Wh kg^−1^ along with a power density of 2178.9 W kg^−1^. Even at 5 C, the cell sustained these energy and power densities, showcasing remarkable performance in low‐temperature environments.

Similarly, high entropy Mn/Fe‐based P2‐Na_0.67_Mn_0.5_Fe_0.334_Cu_0.045_ Mg_0.014_Ti_0.014_Al_0.014_Zr_0.014_Sn_0.014_O_2_ (Mn ^_^ Fe ^_^ HEO) was found by Wang et al.^[^ ^78^ ^]^ As shown in Figure 4b, Mn‐Fe‐LEO undergoes the P2‐P2+OP4‐P2 + P’2 phase transition, and under the same conditions, the high‐entropy component Mn‐Fe‐HEO undergoes the P2‐P2 + OP4‐P2 phase transition, which significantly inhibits the P2‐P’2 phase transition and further enhances the structural stability. In addition, it can be clearly seen from Figure 4c that the redox pairs of high‐entropy components are still active and are divided into three regions during charging and discharging: 1.5–2.8 V (Mn^3+^/Mn^4+^ redox reactions), 2.8–4 V (Fe^3+^/Fe^4+^ and Cu^2+^/Cu^3+^ redox reactions) and 4–4.3 V (O^2−^/(O_2_)^n−^ redox reactions). Simultaneously, introducing inert elements (Mg, Ti, Al, Zr, and Sn) into the high‐entropy structure does not reduce capacity; instead, it significantly enhances reversible capacity. The Mn‐Fe‐HEO cathode delivers 113.7 mAh g^−1^ at 1 C across 1.5–4.3 V and 195.1 mAh g^−1^ at 0.05 C. Zhu et al. incorporated Cr, Fe, Mn, V, and Al into NASICON (HE‐NASICON) to enable multivalent ion doping. Cr, Fe, Mn, and V improve charge compensation and capacity, while Al stabilizes the structure and minimizes Mn‐related Jahn–Teller effects.^[^ ^79^ ^]^ HE‐NASICON undergoes high‐voltage multi‐electron redox reactions (Figure 4d), including V, Fe, Mn, and Cr. Cr^3+^/Cr^4+^ and Mn^3+^/Mn^2+^ have high redox potentials, while Fe^2+^/Fe^3+^ is lower, broadening the voltage range. Optimizing the element ratio improves the material's discharge voltage and energy density. The HE‐NASICON electrode achieves a reversible capacity of 165 mAh g^−1^ at 0.1 C and outperforms Na_4_MnCr (PO_4_)_3_ across 0.1–20 C (1 C = 120 mAh g^−1^). It retains 70.5% capacity after 2000 cycles at 10 C (1.2 A g^−1^). A full cell paired with hard carbon delivers an initial discharge capacity of 131.09 mAh g^−1^, an average output voltage of 3.07 V, and an energy density of 309.5 Wh kg^−1^. Similarly, Hao et al. synthesized HE‐V1.6 using a sol–gel method.^[^ ^80^ ^]^ This high‐entropy sodium vanadium phosphate cathode enables stable V^5+^/V^4+^ redox reactions and maintains structural integrity during multi‐electron transfers. HE‐NVP delivers an energy density of 120 mAh g^−1^ at 5 A g^−1^ and retains 93.1% of its capacity after 2000 cycles (Figure 4e,f), demonstrating excellent long‐term stability.

**Figure 5:**
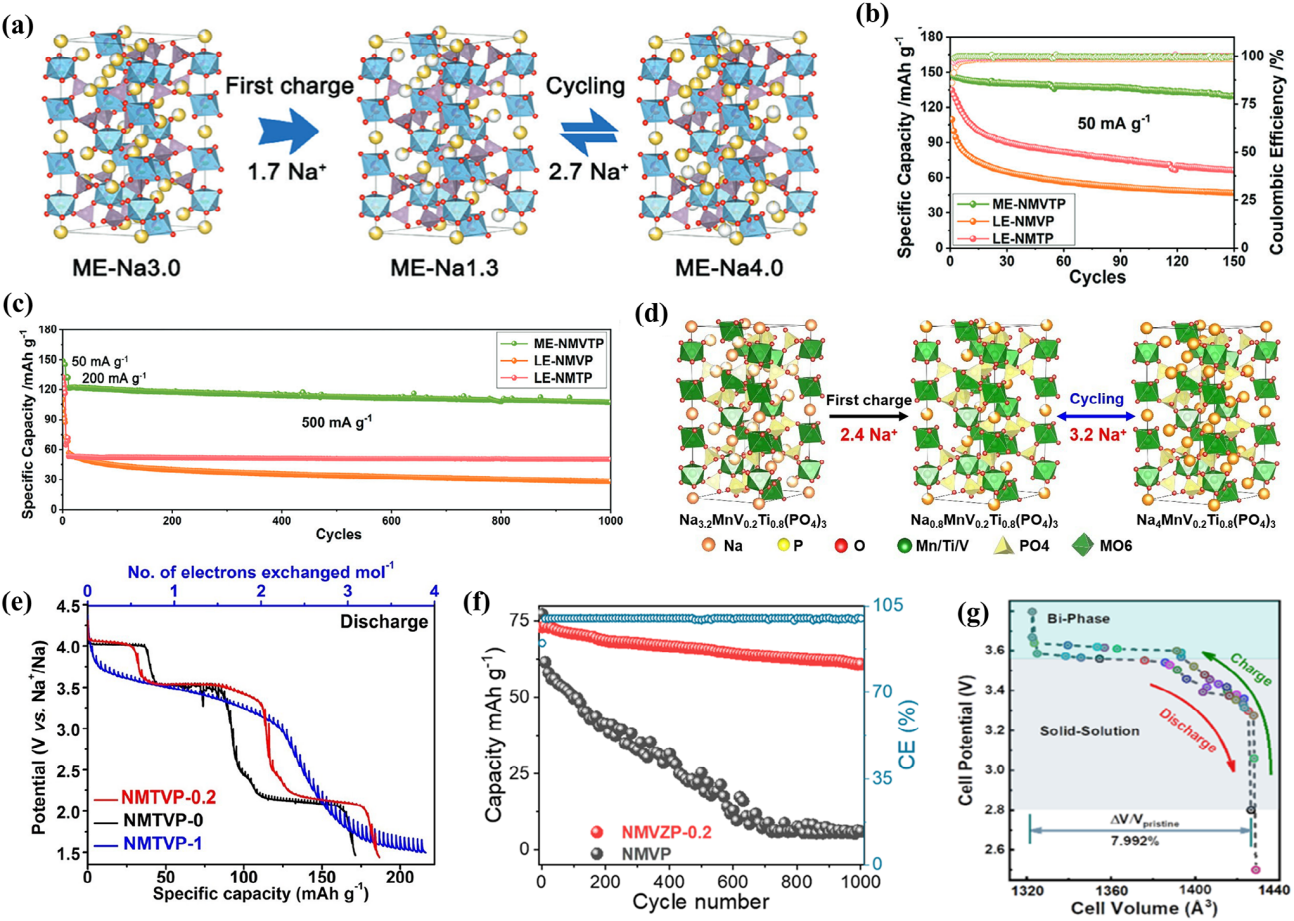
a) Schematic structural change of ME‐NMVTP during charge/discharge. b) Specific capacity and Coulombic efficiency of as‐prepared cathodes at 50 mA g^−1^. c) Long cycling performance of as‐prepared cathodes at 500 mA g^−1^. Reproduced with permission.^[^ ^8^ ^]^ Copyright 2023 Wiley‐VCH GmbH. d) schematic structural change of NMTVP‐0.2 during charge/discharge. e) GITT curves of NMTVP‐1, NMTVP‐0.2, and NMTVP‐0. Reproduced with permission.^[^ ^81^ ^]^ Copyright 2023 Wiley‐VCH GmbH. f) Comparison of long‐term cycling performance between NMVP and NMVP‐0.2. Reproduced with permission.^[^ ^82^ ^]^ Copyright 2023 Elsevier B.V. g) Lattice parameter variation of the NVAMP electrode at different cell potentials. Reproduced with permission.^[^ ^83^ ^]^ Copyright 2021 American Chemical Society.

Researchers have explored the medium‐entropy approach to enhance cathode materials’ electrochemical performance. Zhu et al. synthesized medium‐entropy NASICON compounds,

Na_3_Mn_2/3_V_2/3_Ti_2/3_(PO_4_)_3_/C@CNTs (ME‐NMVTP), using the sol–gel method.^[^ ^8^ ^]^ By incorporating Mn, V, and Ti in a medium‐entropy crystal state, they improved Na^+^ migration. ME‐NMVTP enabled a continuous redox reaction at high voltages up to 4.3 V, facilitating the reversible intercalation of 2.7 Na^+^. As shown in **Figure** **5**a–c, it achieved a reversible capacity of 147.9 mA h g^−1^ at 50 mA g^−1^ and maintained stability over 1000 cycles at 500 mA g^−1^. Similarly, Hu et al. developed medium‐entropy NASICON material Na_3.2_MnTi_0.8_V_0.2_(PO_4_)_3_, which exhibited a fully reversible 3.2‐electron redox reaction. Figure 5d illustrates its structural changes during charge–discharge cycles.^[^ ^81^ ^]^ The five redox pairs, Ti^3+/4+^ (≈2.1 V), V^3+/4+^ (≈3.4 V), Mn^2+/3+^ (≈3.6 V), Mn^3+/4+^ (≈4 V), V^4+/5+^ (≈4.1 V), Figure 5e, contributed to a high discharge capacity of 186.7 mAh g^−1^ and an energy density of 527.2 Wh kg^−1^ across 1.5–4.3 V. Additionally, Zr^4+^, with its larger ionic radius and higher valence state, expanded Na⁺ migration pathways and created vacancies, further improving ionic transport. Thus promoting the kinetics of insertion/extraction electrode reaction of Na^+^, Tang, and others prepared medium‐entropy Na_3.8_MnV_0.8_Zr_0.2_(PO_4_)_3_ (NMVZP‐0.2).^[^ ^82^ ^]^ They employed Zr substitution to improve the reversibility and activity of the redox reactions of V^3+^/V^4+^ and Mn^2+^/Mn^3+^ in the electrode. As a result, the NMVZP‐0.2 electrode exhibited superior performance, achieving a capacity of 73.1 mAh g^−1^ at 20 C, which significantly surpassed the NMVP electrode that only reached 29.7 mAh g^−1^. When the current density increased to 40 and 60 C, the capacity retention of the NMVZP‐0.2 electrode was 80.5% and 77.5%, respectively, while the NMVP electrode failed completely (Figure 5f), thus demonstrating the superiority of medium‐entropy doping. In addition, since the element Mn enhances the stability of the cell structure, Xu and others obtained Na_4_V_0.8_Al_0.2_Mn(PO_4_)_3_ (NVAMP) by selectively substituting vanadium instead of manganese in the Na_4_VMn(PO_4_)_3_ system by choosing the three TM ions, Mg^2+^, Al^3+^, and Ti^4+^ ions.^[^ ^83^ ^]^ This selective substitution reduces the kinetic hysteresis and undesirable structural degradation caused by Mn. Moreover, Al doping helps to reduce Na^+^ transport barriers and suppress Jahn–Teller distortion, improving Na^+^ kinetics and structural stability, thus enhancing electrochemical performance. Experimental results and DFT calculations together confirm that Al‐doped Na_4_V_0.8_Al_0.2_Mn(PO_4_)_3_ cathodes show excellent electrochemical behavior with highly reversible structural evolution and only 8% total volume change (Figure 5g). The NVAMP exhibits a discharge capacity of 84 mA h g^−1^ at 40 C and maintains 92% capacity retention after 1000 cycles at 5 C, within a voltage range of 2.5 to 3.8 V.

**Figure 6:**
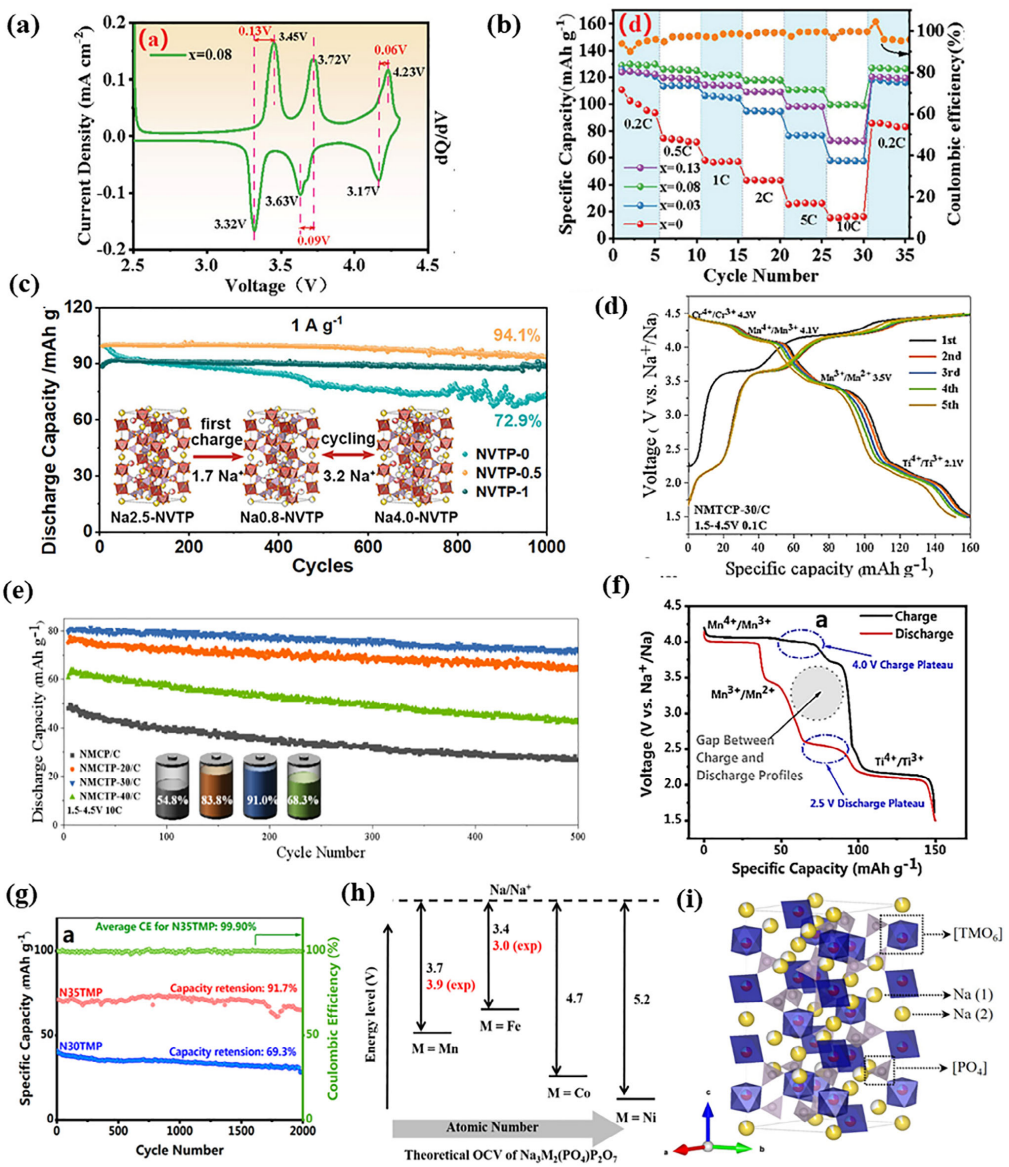
a) Cyclic voltammetry curves of N_1.92_K_0.08_VPF/C sample at a scan rate of 0.1 mV s^−1^. b) Cycling performance of N_1−x_K_x_VPF/C (*x* = 0, 0.03, 0.08, and 0.13) samples at 10 C. Reproduced with permission.^[^ ^84^ ^]^ Copyright 2024 Wiley‐VCH GmbH. c) Schematic structural change of NVTP‐0.5 during charge/discharge and cycling performance at 1 A g^−1^. Reproduced with permission.^[^ ^72^ ^]^ Copyright 2024 American Chemical Society. d) Charge/discharge curves of the initial five cycles for NMTCP‐30/C. e) Cycling performance of Na_4−2x_Mn_1−x_Ti_x_Cr(PO_4_)_3_ (*x* = 0, 0.2, 0.3, and 0.4) at 10 C. Reproduced with permission.^[^ ^85^ ^]^ Copyright 2024 Elsevier Inc. f) GCD profiles of N35TMP at 0.1 C within the voltage range between 1.5 and 4.2 V. g) Cycling performances of N30TMP and N35TMP. Reproduced with permission.^[^ ^86^ ^]^ Copyright 2021 American Chemical Society. h) The theoretical working potential of Na_3_M_2_(PO_4_)P_2_O_7_ (M = Fe, Mn, Co, and Ni) cathode materials versus Na/Na^+^ for SIBs. Reproduced with permission.^[^ ^34^ ^]^ Copyright 2021 Elsevier B.V. i) Crystal structure model of NVMTP. Reproduced with permission.^[^ ^87^ ^]^ Copyright 2023 Elsevier B.V.

Low‐entropy strategies can enhance a material's operating voltage and energy density by selecting and optimizing suitable doping elements like K, Ni, and Ti. Wu et al. synthesized K‐doped Na_0.92_K_0.08_V_2_(PO_4_)_2_F_3_/C by partially substituting Na^+^ with K^+^ in NVPF crystals.^[^ ^84^ ^]^ K⁺ doping improves the cathode's electronic conductivity by reducing the band gap without altering the V valence state. As shown in **Figure** **6**a, the material exhibits three voltage plateaus at 3.4, 3.7, and 4.2 V, corresponding to Na^+^ insertion/extraction via the V^3+/4+^ redox pair. Additionally, K^+^ doping expands Na^+^ migration channels, enabling a highly reversible phase transition that enhances cycling performance. As shown in Figure 6b, the discharge capacity at 0.2 C, 0.5 C, 1 C, 2 C, 5 C, and 10 C is 128.8, 126.1, 122.1, 117.1, 110.6, and 99.6 mAh g^−1^, respectively. The material retains 92% capacity after 100 cycles at 0.2 C and 60.2% after 5000 cycles at 10 C, maintaining a capacity of 113.3 mAh g^−1^. In additionally, Na_2.5_V_1.5_Ti_0.5_(PO_4_)_3_/C (NVTP‐0.5) was obtained by spray drying assisted annealing by Zhu and co‐workers.^[^ ^72^ ^]^ NVTP‐0.5 combines the multiple redox properties of V, the good stability of Ti, and the synergistic contribution of biphasic metals of appropriate composition. As shown in Figure 6c, the NVTP‐0.5 cathode achieves a high discharge capacity of 192.42 mAh g^−1^ (corresponding to 3.2 electron transfers) and an energy density of 497.3 Wh kg^−1^, thanks to the redox pairs V^2+^/V^3+^, V^3+^/V^4+^, and Ti^3+^/Ti^4+^. It maintains excellent stability, with 94.1% capacity retention after 1000 cycles at 1 A g^−1^, and performs reliably even in extreme temperatures (50 and −15 °C). Low‐entropy doping further enhances structural stability and electronic conductivity, particularly at high voltages. Based on this principle, Chen et al. incorporated Ti^4+^ into NASICON‐type Na_4_MnCr(PO_4_)_3_ using the sol–gel method, creating Na_3.4_Mn_0.7_Ti_0.3_Cr(PO_4_)_3_/C material.^[^ ^85^ ^]^ Since Ti^4+^ has a similar ionic radius to Mn^2+^ and a d° configuration, substituting it for Mn^2+^ shortens TM─O bond lengths without disrupting the original structure, stabilizing the TMO_6_ octahedra. This Ti doping enhances energy density, as shown in Figure 6d. The Na_3.4_Mn_0.7_Ti_0.3_Cr(PO_4_)_3_/C cathode undergoes Mn^2+^/Mn^3+^ (3.5 V), Mn^3+^/Mn^4+^ (4.1 V), Cr^3+^/Cr^4+^ (4.3 V), and Ti^3+^/Ti^4+^ (2.1 V) redox reactions, transferring ≈2.86 electrons. It retains 91% capacity after 500 cycles at 10 C (1.5–4.5 V) and delivers 99.5 mAh g^−1^ at 5 C, achieving an energy density of 541.6 Wh kg^−1^ (Figure 6e). Elemental doping optimization also improves electrochemical performance by adjusting Na content. Zhang et al. added 16.7% more Na than the stoichiometric amount to Na_3_TiMn(PO_4_)_3_ (N35TMP), reducing Mn^2+^ occupancy in the M1 site.^[^ ^86^ ^]^ This suppressed Na^+^/Mn^2+^ cation mixing, stabilizing the structure, preventing a 2.5 V plateau, and increasing discharge capacity in the high‐voltage region. As shown in Figure 6f, the N35TMP material suppresses the 2.5 V discharge plateau, resulting in a voltage change within the range of 1.5–4.2 V. This involves three redox pairs: Mn^4+^/Mn^3+^ at 4 V, Mn^3+^/Mn^2+^ at 3.5 V, and Ti^4+^/Ti^3+^ at 2.1 V (vs Na^+^/Na). This shift increases the average discharge voltage of N35TMP from 3.41 to 3.65 V, achieving a Na^+^ storage capacity of 101.5 mAh g^−1^ at 0.1 C, and an energy density of 370 Wh kg^−1^. It also retains 91.7% capacity after 2000 cycles at 2 C, significantly better than the original N30TMP (69.3% retention, Figure 6g). In elemental doping, the cost is an important factor. Researchers often select low‐cost elements to replace expensive metals like vanadium. For example, Wang et al. used manganese (Mn) to enhance the performance of Na_3_Fe_2_(PO_4_)(P_2_O_7_) (NFFPP).^[^ ^34^ ^]^ By doping with Mn, NFMPP, the average working potential increased from 3.05 to 3.27 V (vs Na/Na^+^), and the carbon‐coated NFMPP material showed an initial reversible capacity of 105 mAh g^−1^ at 0.1 C. In full‐cell configuration with hard carbon, the NFMPP‐HC cell delivered an initial capacity of 54 mAh g^−1^ at 2 C and retained 81% after 150 cycles. Mn doping improves electrochemical performance by facilitating a reversible redox process between Fe^3+^/Fe^2+^ and Mn^3+^/Mn^2+^ (Figure 6h). However, excessive Mn doping negatively impacts performance. A balanced doping strategy, such as the use of Ti^4+^ and Mn^2+^ to obtain the Na_3_VMn_0.5_Ti_0.5_(PO_4_)_3_/C (NVMTP/C), leads to excellent results.^[^ ^87^ ^]^ The NVMTP/C material undergoes a three‐electron reaction, thanks to the synergy between vanadium, manganese, and titanium, resulting in an average operating voltage of 3.4 V and a stable capacity of 160.3 mAh g^−1^. It also performs well at high current densities, with a capacity of 82 mAh g^−1^ at 17 C. When paired with an HC anode in a full cell, NVMTP/C delivers a high initial discharge capacity of 145 mAh g^−1^ and an energy density of 485 Wh kg^−1^ at 3.35 V (Figure 6i).

#### 3.1.2 Anionic Doping

Anion doping, like cation doping, is an effective strategy to enhance the electrochemical performance of polyanion electrode materials. By substituting anions, structural changes during charge and discharge can be minimized, improving cycling stability and safety.

**Figure 7:**
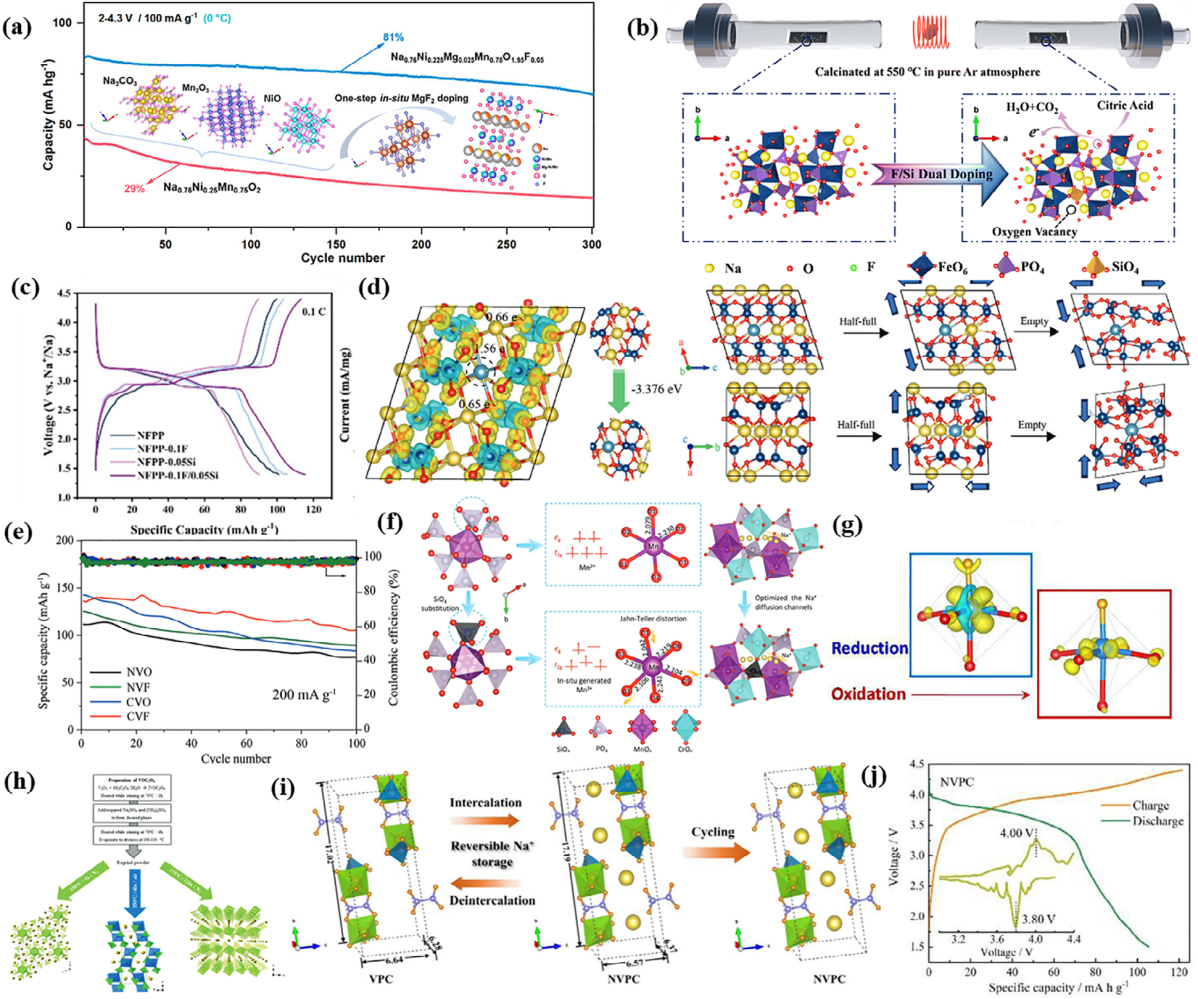
a) The changes of situ Mg@Ni/F−doped samples crystal structure and cycling performance between 2 to 4.3 V at 100 mA g^−1^. Reproduced with permission.^[^ ^33^ ^]^ Copyright 2024 American Chemical Society. b) Schematic illustration of the oxygen vacancy formation via F/Si dual doping in Na_4_Fe_3_(PO_4_)_2_P_2_O_7_. c) Galvanostatic charge–discharge profiles of all four materials at 0.1 C. Reproduced with permission.^[^ ^88^ ^]^ Copyright The Royal Society of Chemistry 2024. d) Structure transformation of CVF under different charging states. e) Cyclic performance at 200 mA g^−1^. Reproduced with permission.^[^ ^35^ ^]^ Copyright 2024 Wiley‐VCH GmbH. f) Schematic diagram illustrating the optimization of the Na^+^ diffusion channels in Na_4_MnCr(PO_4_)_2.9_(SiO_4_)_0.1_ cathodes by SiO_4_ substitution. Reproduced with permission.^[^ ^91^ ^]^ Copyright 2023 Wiley‐VCH GmbH. g) The f^−^ and f^+^ Fukui functions computed for these two processes confirm the expected orbital shape. Reproduced with permission.^[^ ^60^ ^]^ Copyright 2016 American Chemical Society. h) A schematic illustrated the facile synthesis of Na–V–S–O phases. Reproduced with permission.^[^ ^92^ ^]^ Copyright The Royal Society of Chemistry 2018. i) Schematic illustration of the sodium storage mechanism and stability of the layered framework. j) Galvanostatic charge/discharge curves and their corresponding dQ/dV patterns of NVPC.^[^ ^93^ ^]^ Reproduced with permission.^[^ ^93^ ^]^ Copyright 2022 Elsevier B.V.

Fluorination is a widely used anion doping method because fluoride (F^−^) has a stronger affinity than oxide (O^2−^). This helps stabilize metal ions, reducing unwanted side reactions at the electrode interface. A stable metal–F bond enhances structural integrity under high voltage conditions.^[^ ^88^ ^]^ For example, He et al. synthesized P2‐Na_0.76_Ni_0.225_Mg_0.025_Mn_0.75_O_1.95_F_0.05_ by partially replacing O with F and Ni with cost‐effective Mg.^[^ ^33^ ^]^ This optimized material improved capacity and stability by enhancing Ni^2+^/Ni^4+^ and oxygen‐reducing activities. It delivered a high capacity of 132.9 mAh g^−1^ at an average voltage of 3.48 V, compared to 114.9 mAh g^−1^ and 3.32 V for the undoped material, increasing the operating voltage by 0.16 V. Within 2–4.3 V, it retained 83% of its capacity after 150 cycles at 100 mA g^−1^. When charged to 4.5 V, it achieved 133.1 mAh g^−1^ with a discharge potential of 3.55 V (vs Na/Na⁺), maintaining 72.8% retention after 100 cycles (**Figure** **7**a).

F/Si double‐doped Na_4_Fe_3_(PO_4_)_2_P_2_O_7_ (NFPP‐0.1F/0.05Si) to enhance Na storage performance investigated by Gao et al.^[^ ^88^ ^]^ By introducing oxygen vacancies, F^−^ improved conductivity, while the strong Si–O tetrahedra reinforced structural stability. A schematic of this doping effect is shown in Figure 7b. The doped NFPP cathode delivered a capacity of 119.6 mAh g^−1^ at 0.1 C (1.4–4.4 V), a rate performance of 67.7 mAh g^−1^ at 10 C, and 80.86% capacity retention at 50 C, demonstrating excellent cycling stability (Figure 7c). Similarly, Han et al. enhanced Na_2_CaV_2_O_6_F (CVF) by Ca/F co‐doping, increasing Na‐layer spacing and reducing crystal water content, which minimized volume shrinkage.^[^ ^35^ ^]^ This tuning lowered the Na^+^ diffusion barrier compared to undoped Na_2_V_2_O_6_ (NVO). As shown in Figure 7d–f, despite some structural contraction during charge/discharge, the doping stabilized the framework. The material achieved 138 mAh g^−1^ at 200 mA g^−1^ and maintained 104.88 mAh g^−1^ after 100 cycles (1.5–4 V). In a full‐cell configuration with an HC anode, CVF delivered an initial discharge capacity of 65 mAh g^−1^ at 400 mA g^−1^. However, anion doping is not always beneficial. While it enhances some properties, it may negatively affect others. To optimize performance, fluorine doping is often combined with other strategies, such as partial PO_4_^3−^ substitution or introducing vanadium vacancies to improve Na^+^ migration.^[^ ^61^ ^]^

Anion substitution is a key strategy for enhancing the electrochemical performance of polyanion cathode materials. Replacing PO_4_^3−^ with anions such as BO_4_^3−^, SiO_4_^4−^, SO_4_^2−^, and (C_2_O_4_)^2−^ improves conductivity, lowers the Na⁺ diffusion barrier, and enhances structural stability. Boron (B) doping, for instance, modifies the electron density of nearby O^2−^ anions, affecting Na^+^ transport.^[^ ^89^ ^]^ Xiao et al. found that B doping raises the oxidation state of V atoms, increasing operating voltage.^[^ ^90^ ^]^ The V–O–B electronic bridges formed due to strong covalent interactions improve both structural stability and lattice contraction. Similarly, replacing PO_4_^3−^ with SiO_4_^4−^ in Na_4_MnCr (PO_4_)_2.9_(SiO_4_)_0.1_ optimized Na^+^ diffusion and enhanced Mn oxidation states, improving insertion/extraction kinetics.^[^ ^91^ ^]^ The SiO_4_^4−^ substitution increased the redox activity of Mn^2+^/Mn^3+^, leading to a higher energy density and an initial discharge capacity of 149.9 mAh g^−1^ at 0.1 C (1.5–4.5 V). SO_4_^2−^ substitution also improves performance. Sun et al. reported Na_2_VO(SO_4_) *_x_* cathodes with a high 4.5 V operating voltage based on the V^4+^/V^5+^ redox reaction.^[^ ^60^ ^]^ The inductive effect of SO_4_^2−^ increased redox potential by 0.6 V compared to PO_4_^3−^. Figure 7g illustrates the orbital interactions stabilizing these voltage gains.^[^ ^60^ ^]^ Driscoll et al. synthesized sodium vanadyl sulfate compounds NaV (SO_4_)_2_, Na_2_VO(SO_4_)_2_, and Na_3_V(SO_4_)_3_ using a simple, low‐temperature (≤400 °C) method, reducing production costs.^[^ ^92^ ^]^ These materials, depicted in Figure 7h, feature quasi‐lamellar or 3D structures that facilitate Na^+^ transport. Beyond monoanionic substitution, polyanionic coupling further enhances performance. Li et al. synthesized Na_2_(VO)_2_(HPO_4_)_2_(C_2_O_4_) (NVPC), combining (C_2_O_4_)^2−^ and (HPO_4_)^2−^ to stabilize V^4+/5+^ redox centers at ≈4 V.^[^ ^93^ ^]^ As shown in Figure 7i, NVPC exhibited 121.5 mAh g^−1^ at 0.1 C and retained 61.2% capacity over 1000 cycles at 5 C (Figure 7j). These findings highlight anion substitution as a versatile strategy for improving cathode materials in SIBs.

To sum up, elemental doping is a useful technique for raising operating voltage, improving cycle stability, and increasing energy density in SIB cathode materials, all of which increase electrochemical performance (**Table** **1**). On the other hand, oxygen loss, phase transitions, and structural instability brought on by high‐voltage cycling might impair long‐term performance. By increasing resistance to volume fluctuations, optimizing the material's structure, for example, by modifying particle size and layer spacing, helps lessen these problems. Doping with metals like as magnesium and aluminum improves cycle life and further stabilizes the structure. Doping is a viable approach for high‐voltage SIBs as it also enhances electrical conductivity and Na^+^ transport by adding electrochemically active metals and non‐metallic components.

**Table 1:** Summary of doping elements, redox‐active couples, and voltage ranges for various cathode materials in SIBs.

Materials	Crystal type	Voltages[V]	Redox couples	Electrolyte	Rate capacity	Cycling stability	Refs.
Na_3_Mn_2/3_V_2/3_Ti_2/3_(PO_4_)_3_/C@CNTs	NASICON	1.5–4.3	–	1 m NaClO_4_ in PC + 5% FEC	147.9 Ah g^−1^ at 50 mA g^−1^	Retained 88.3% after 1000 cycles at 500 mA g^−1^	[8]
Na_3.4_Mn_0.7_Ti_0.3_Cr(PO_4_)_3_/C	NASICON	2.1–4.3	Mn^2+/3+^ at 3.5 V, Mn^3+/4+^ at 4.1 V, Cr^3/+4+^ at 4.3 V, Ti^3+/4+^ at 2.1 V	1 m NaClO_4_ in PC+ 5% FEC	99.5 mA h g^−1^ at 5 C	Retained 91% after 500 cycles at 10 C	[94]
Na_2_(VO)_2_(HPO_4_)_2_(C_2_O_4_)	NASICON	1.5–4.4	V^4+/5+^ at 4/3.8 V	1 m NaClO_4_ in PC/EC (1:1) +5 vol% FEC	105 Ah g^−1^ at 0.1 C	Retained 61.2% after 1000 cycles at 5 C	[93]
Na_3.32_V_1.6_Cr_0.08_Fe_0.08_Mn_0.08_Mg_0.08_Ca_0.08_(PO_4_)_3_	NASICON	1.2–4.2	V^4+/5+^ at 4 V	1 m NaPF_6_ in diglyme	120 mA h g^−1^ at 5 A g^−1^	Retained 93.1% after 2000 cycles at 5 A g^−1^	[80]
Na_0.92_K_0.08_V_2_(PO_4_)_2_F_3_	NASICON	2.5–4.3	V^3+/4+^ at 3.7–4.2 V	1 m NaClO_4_ in PC/EC (1:1) + 5 vol% FEC	128.8 mA h g^−1^ at 0.2 C	Retained 92% after 100 cycles at 0.2 C	[84]
Na_2.95_K_0.05_V_2_(PO_4_)_3_	NASICON	2.5–3.8	V^3+^/V^4+^ at 3.4 V	1 M NaPF_6_ in diglyme	97.8 mA h g^−1^ at 50 C	Retained 78.6% after 500 cycles at 50 C	[95]
Na_3_V_1.9_(Ca, Mg, Al, Cr, Mn)_0.1_(PO_4_)_2_F_3_	NASICON	2–4.3	Mn^2+/3+^ at 3.54 V	1 M NaClO_4_ in PC + 5 vol% FEC	445.42 mA h g^−1^ at 5 C	Retained 80.4% after 2000 cycles at 20 C	[77]
Na_3.2_MnTi_0.8_V_0.2_(PO_4_)_3_	NASICON	1.5–4.5	V^5+/4+^ at 4.1 V, Mn^4+/3+^ at 4 V, Mn^3+/2+^ at 3.6 V, V^4+/3+^ at 3.4 V, Ti^4+/3+^ at 2.1 V	1 M NaClO_4_ in EC/PC (1:1 w/w) + 5% FEC	172.5 mA h g^−1^ at 50 mA g^−1^	Retained 86.4% after 96 cycles at 100 mA g^−1^	[81]
Na_3_Cr_0.5_V_1.5_(PO_4_)_3_	NASICON	1.5–4.2	V^5+/4+^ at 4.02 V, V^4+/3+^ at 3.43 V, V^3+/2+^ at 1.65 V	1 M NaClO_4_ in EC/PC (1:1 v/v) + 5 wt.% FEC	176 mA h g^−1^ at 0.2 C	Retained 67.4% after 1000 cycles at 20 C	[96]
Na_4_V_0.8_Al_0.2_Mn (PO_4_)_3_	NASICON	2.5–3.8	V^3+/4+^ at 3.4 V, Mn^2+/3+^ at 3.6 V	1 M NaClO_4_ in PC+ 2 wt.% FEC	84 mA h g^−1^ at 40 C	Retained 92% after 1000 cycles at 5 C	[83]
Na_3_Fe_1.9_Ni_0.1_(PO_4_) P_2_O_7_/C	NASICON	1.5–4	PO_4_^3−^–P_2_O_7_^4−^ (3.1 V)	–	100.7 mA h g^−1^ at 0.1 C	Retained 77.43% after 2000 cycles at 5 C	[97]
Na_2.5_V_1.5_Ti_0.5_(PO_4_)_3_/C	NASICON	2.5–3.8	Ti^3+/4+^(2.1 V), V^2+^/V^3+^ (1.6 V) V^3+^/V^4+^ (3.4 V)	–	192.42 mA h g^−1^ at 20 mA g^−1^	Retained 94.1% after 1000 cycles at 1 A g^−1^	[72]
Na_3_VMn_0.5_Ti_0.5_(PO_4_)_3_/C	NASICON	1.5–4.15	V^4+/5+^at 3.8 V, V^3+/4+^at 3.4 V, Ti^3+/4+^at 2.1 V, V^2+/3^ at 1.63 V	1 M NaClO₄ in PC	82 mA h g^−1^ at 17 C	Retained 83% after 1000 cycles at 5.67 C	[98]
Na_3.8_MnV_0.8_Zr_0.2_(PO_4_)_3_	NASICON	2–3.8	V^4+/5+^at 3.8 V, Mn^2+/3+^ at 3.59 V	1 M NaClO₄ in EC/PC (1:1 v/v) +5 wt.% FEC	73.1 m Ah g^−1^ at 20 C	Retained 83.5% after 1000 cycles at 2 C	[99]
Na_3.4_Mn_0.7_Ti_0.3_Cr(PO_4_)_3_/C	NASICON	1.5–4.3	Mn^2+/3+^ (3.5 V), Mn^3+/4+^ (4.1 V), Cr^3+/4+^ (4.3 V), Ti^3+/4+^ (2.1 V)	1 M NaClO_4_ in PC + 5 wt.% FEC	99.5 mA h g^−1^ at 5 C	Retained 91% after 500 cycles at 10 C	[94]
Na_3_TiMn(PO_4_)_3_	NASICON	2.5–4.2	Mn^4+/3+^ at 4 V, Mn^3+/2+^ at 3.5 V, Ti^4+/3+^ at 2.1 V	1 M NaPF_6_ in PC + 5 wt.% FEC	101.5 mA h g^−1^ at 0.1 C	Retained 91.7% after 2000 cycles at 2 C	[100]
Na_4_Fe_3_(PO_4_)_2_P_2_O_7_ −0.1F/0.05Si		1.4–4.4	Fe^2+^/Fe^3+^ at 2.99/2.86 and 3.25/3.19 V	1 M NaClO_4_ in PC + 5 wt.% FEC	119.6 mA h g^−1^ at 0.1 C	Retained 80.86% after 4000 cycles at 50 C	[88]
Na_0.76_Ni_0.225_Mg_0.025_Mn_0.75_O_1.95_F_0.05_	P2	2–4.5	Ni^2+^/Ni^4+^ at 3.48 V	1 M NaClO_4_ in PC + 2 wt.% FEC	132.9 mA h g^−1^ at 100 mA g^−1^	Retained 83% after 150 cycles at 100 mA g^−1^	[33]
Na_2_CaV_2_O_6_F		1.5–4	–	1 M NaClO_4_ in EC/DMC/EMC (1:1 v/v) + 5 wt.% FEC	138 mA h g^−1^ at 200 mA g^−1^	Retained 76% after 100 cycles at 200 mA g^−1^	[101]
Na_4_MnCr(PO_4_)_2.9_(SiO_4_)_0.1_	NASICON	1.5–4.5	Mn^2+/3+^ at 3.70/3.45, Mn^3+/4+^ at 4.23/4.10, Cr^3+/4+^ at 4.47/4.36 V	1 M NaClO_4_ in PC+10 wt.%FEC	149.9 mA h g^−1^ at 0.1 C	Retained 80% after 200 cycles at 100 mA g^−1^	[91]

The range of 1 C in the table is ≈90 mA g^−1^ to 1.2 A g^−1^.

Full form for the various abbreviations used in the table is given: EC‐ Ethylene Carbonate, PC‐ propylene carbonate, FEC‐ Fluoroethylene Carbonate, DMC‐ Dimethyl Carbonate; DME‐ Dimethoxy Ethane; NaTFSI‐ Sodium bis (trifluoromethanesulfonyl) imide; SUL‐ Sulfolane (a sulfone‐based solvent); OTE‐ 1H,1H,5H‐octafluoropentyl‐1,1,2,2‐tetrafluoroethyl ether (a non‐solvent diluent).

### 3.2 Carbon Coating

Because of its superior electrical conductivity, chemical stability, and affordability, carbon modification is being researched extensively. When carbon and active materials are combined, an exterior conductive scaffolding is created that improves the flow of electrons and sodium ions while safeguarding the material junction.^[^ ^102^ ^]^ Additionally, carbon reduces strain from volume fluctuations during cycling by acting as an elastic cushion. By guaranteeing quick electron‐ion transport and avoiding interactions with the electrolyte, this arrangement enhances electrochemical performance. Results from investigations employing carbon materials are summarized in **Table** **2**.

**Table 2:** Particle morphology and electrochemical performance of NASICON compounds synthesized using various methods.

Materials	Voltages [V]	Coating composition	Rate capacity	Cycling stability	Refs.
Na_3_V_2_(PO_4_)_2_F_3−y_O_z_/MCLC@C	2.5–4.3	MCLC + Citric Acid	117.3 mA h g^−1^ at 0.1 A g^−1^	Retained 80% after 1100 cycles at 2 A g^−1^	[103]
Na_3_V_2_(PO_4_)_3_	2.3–4	NSF‐CDs	88.9 mA h g^−1^ at 200 C	Retained 90.2% after 5000 cycles at 100 C	[104]
Na_3_Mn_0.5_V_0.5_Ti(PO_4_)_3_/rGO	1.5–4.3	rGO	284.6 mA h g^−1^ at 0.1 C	Retained 76.2% after 500 cycles at 1 C	[105]
Na_3_Mn_2/3_V_2/3_Ti_2/3_(PO_4_)_3_/C@CNTs	1.5–4.3	C@CNTs	147.9 mA h g^−1^ at 50 mA g^−1^	Retained 88.3% after 1000 cycles at 500 mA g^−1^	[8]
Na_0.92_K_0.08_V_2_(PO_4_)_2_F_3_/C	2.5–4.3	C@CNTs	128.8 mA h g^−1^ at 0.2 C	Retained 92% after 100 cycles at 0.2 C	[84]
Na_2.5_V_1.5_Ti_0.5_(PO_4_)_3_/C	1.5–4	C@CNTs	192.42 mA h g^−1^ at 20 mA g^−1^	Retained 94.1% after 1000 cycles at 1 A g^−1^	[72]
Na_3.4_Mn_0.7_Ti_0.3_Cr(PO_4_)_3_/C	1.5–4.5	C@CNTs	159 mA h g^−1^ at 0.1 C	Retained 91% after 500 cycles at 10 C	[85]
Na_3_VMn_0.5_Ti_0.5_(PO_4_)_3_/C	1.5–4.15	C	82 mA h g^−1^ at 17 C	Retained 95% after 3000 cycles at 11.34 C	[87]
Prussian Blue@Graphene Oxide‐Polyvinylpyrrolidone (PB@GO‐PVP)	2–4.2	Graphene	165.2 mAh g^−1^ at 1 C	Retained 72.7% after 500 cycles at 1 C	[108]
NaLi_0.2_Mn_0.8_O_2_(NLM@C)	1.5–4	C	160 mAh g^−1^ at 0.1 C	Retained 62.5% after 100 cycles at 1 C	[106]
AgHCF@CNT	1–4	@CNT	90.7 mAh g^−1^ at 2 A g^−1^	Retained 74% after 500 cycles at 0.5 A g^−1^	[107]

**Figure 8:**
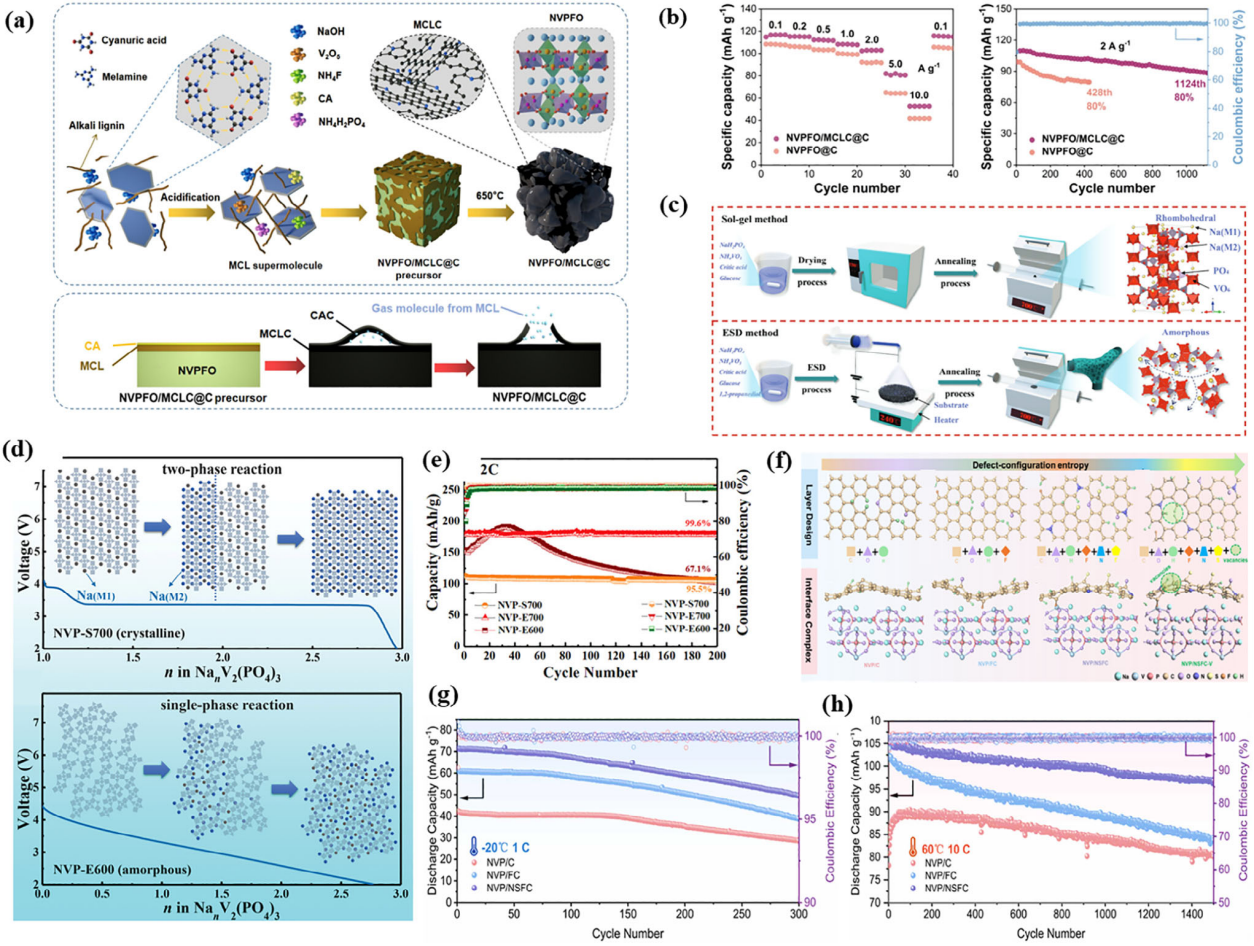
a) Schematic illustration of the synthetic processes of NVPFO@MCLC and diagram of the process in which the MCLC carbon layer. b) Rate performance and cycling performance of NVPFO@C and NVPFO/MCLC@C at 2 A g^−1^.^[^ ^103^ ^]^ Reproduced with permission.^[^ ^103^ ^]^ Copyright 2024 Elsevier B.V. c) Schematic illustration of the synthesis processes of Na_3_V_2_(PO_4_)_3_ by sol–gel and ESD methods. d) Schematic diagram of voltage curve and corresponding internal process of crystalline NVP and amorphous NVP, the small grown and purple circles represent Na atoms, and the gray squares stand for the lattice. e) Cycle performance of the NVP‐S700, NVP‐E700, and NVPE600 electrodes. Reproduced under terms of the CC‐BY license.^[^ ^71^ ^]^ Copyright 2022, The Authors, published by Wiley‐VCH GmbH. f) Carbon layer models with different doping elements and Composite models of the four designed carbon layers with NVP cathodes. g) Low‐temperature performance of NVP/C series samples at 20 °C. h) High‐temperature performance of NVP/C series samples at 60 °C. Reproduced with permission.^[^ ^104^ ^]^ Copyright 2024 Elsevier Ltd.

Carbon modification is an effective strategy to enhance the operating voltage and electrochemical performance of Na^+^ cathode materials. Ma et al. used a sol–gel method with melamine‐cyanate‐lignin (MCL) and citric acid (CA) as carbon sources to create a dual‐carbon cladding structure in Na_3_V_2_(PO_4_)_2_F_3−y_O_z_ (NVPFO).^[^ ^103^ ^]^ This structure included an external coral‐like carbon layer and an internal nitrogen‐doped carbon layer (**Figure** **8**a). The carbon coating improves conductivity by forming a conductive network that accelerates electron transport and reduces Na^+^ diffusion resistance. This facilitates faster Na^+^ insertion/extraction, improving rate performance. The NVPFO material achieved a specific capacity of 117.3 mAh g^−1^ at 0.1 A g^−1^ and maintained 80% capacity retention after 1100 cycles at 2 A g^−1^ (Figure 8b). These results highlight carbon doping as a promising approach for improving the efficiency and longevity of sodium‐ion cathode materials. Ma et al. synthesized 3D porous Na_3_V_2_(PO_4_)_3_ (NVP) using electrostatic spray deposition (ESD) on a melamine‐based carbon foam (CF) substrate.^[^ ^71^ ^]^ By adjusting the crystallinity through calcination at different temperatures, they activated the V^5+^/V^4+^ redox pair, enabling a three‐electron reaction for Na storage. As shown in Figure 8c, NVP‐E700 and NVP‐E600 were obtained by sintering at 700 and 600 °C, respectively, while NVP‐S700 was synthesized using the conventional sol–gel method. Figure 8d illustrates how the disordered NVP structure facilitates simultaneous V^4+^/V^3+^ and V^5+^/V^4+^ redox reactions, achieving an ultra‐high specific capacity of 179.6 mAh g^−1^ at 0.2 C. In contrast, unmodified NVP, relying only on the V^4+^/V^3+^ redox pair (3.4 V v Na^+^/Na), has a theoretical capacity of 117.6 mAh g^−1^ (Figure 8e). To address the weak bonding between the carbon layer and NVP, Li et al. introduced defect‐configuration entropy by doping five heteroatoms (H, O, N, S, and F) and creating carbon vacancies.^[^ ^104^ ^]^ As shown in Figure 8f, this engineered a variety of non‐homogeneous carbon layers combined with NVP to form NVP/NSFC composite cathodes. This material achieved a high specific capacity of 88.9 mAh g^−1^ at 200 C, with 90.2% capacity retention after 5000 cycles at 100 C. Furthermore, as shown in Figure 8g,h, NVP/NSFC maintained 69.6% capacity after 300 cycles at −20 °C (1 C) and 92.9% capacity after 1500 cycles at 60 °C (10 C). In a full cell paired with an HC anode, the NVP/NSFC‖HC system retained 93.1% of its capacity after 1400 cycles at 5 C, demonstrating excellent electrochemical stability across a wide temperature range. A NASICON‐structured Na_3_Mn_0.5_V_0.5_Ti(PO_4_)_3_ (NMVTP) cathode bonded with reduced graphene oxide (rGO) was developed by Liu et al.^[^ ^105^ ^]^ Its ultrafine nanosheet structure enhances electron transfer, stabilizes the electrode, and prevents dissolution. NMVTP/rGO exhibits excellent electrochemical performance, delivering 284.6 mAh g^−1^ at 0.1 C and 108.5 mAh g^−1^ at 5 C, with 76.2% capacity retention after 500 cycles at 1 C. Quyen et al. synthesized a carbon‐coated sodiumlithiummanganese oxide (NLM@C) via a traditional solid‐state reaction method.^[^ ^106^ ^]^ The carbon coating reduces the resistance of the NLM oxide, increasing its rate capability. The modified material shows a specific capacity of 160 mAh g^−1^ at 0.1 C and 115 mAh g^−1^ at 1 C. In addition, Zhao et al. synthesized vacancy‐free and anhydrous silver hexacyanoferric oxide (AgHCF) nanoparticles by co‐precipitation and prepared AgHCF@CNTs composites by combining them with carbon nanotubes (CNTs).^[^ ^107^ ^]^ This material is capable of realizing a four‐electron transfer reaction as the cathode material of SIBs, which significantly improves the electrochemical performance of the batteries. They found that the four‐electron transfer reaction enabled the material to have a higher theoretical capacity of 185 mAh g^−1^. In practical tests, AgHCF@CNTs exhibited a reversible capacity of 168.4 mAh g^−1^ at a current density of 50 mA g^−1^, which is much higher than that of conventional PBA materials. It also exhibits great multiplicative performance and cycling stability, maintaining a capacity of 90.7 mAh g^−1^ even at a high current density of 2 A g^−1^ and retaining 74% of the capacity after 500 cycles.

Carbon coating is a highly effective strategy for enhancing the electrochemical performance of Na^+^ cathode materials. By forming a conductive network, it accelerates electron transfer, reduces Na^+^ diffusion resistance, and stabilizes the electrode structure. Advanced techniques, such as dual‐carbon cladding and defect‐engineered carbon layers, further improve capacity retention and cycling stability.

### 3.3 Modifying the Synthesis Method

The precise tuning of the synthesis method is crucial to the performance of the cathode material, which not only determines the electrochemical properties of the battery but also directly affects key factors such as energy density, structural stability, electronic conductivity, and ion diffusion kinetics. The well‐designed synthesis strategy can precisely control the particle size and morphology of cathode materials, and the optimization of the synthesis process can help to reduce the defects of the materials and enhance the structural stability, which is crucial for the long cycle life and safety of batteries under high voltage conditions.

**Figure 9:**
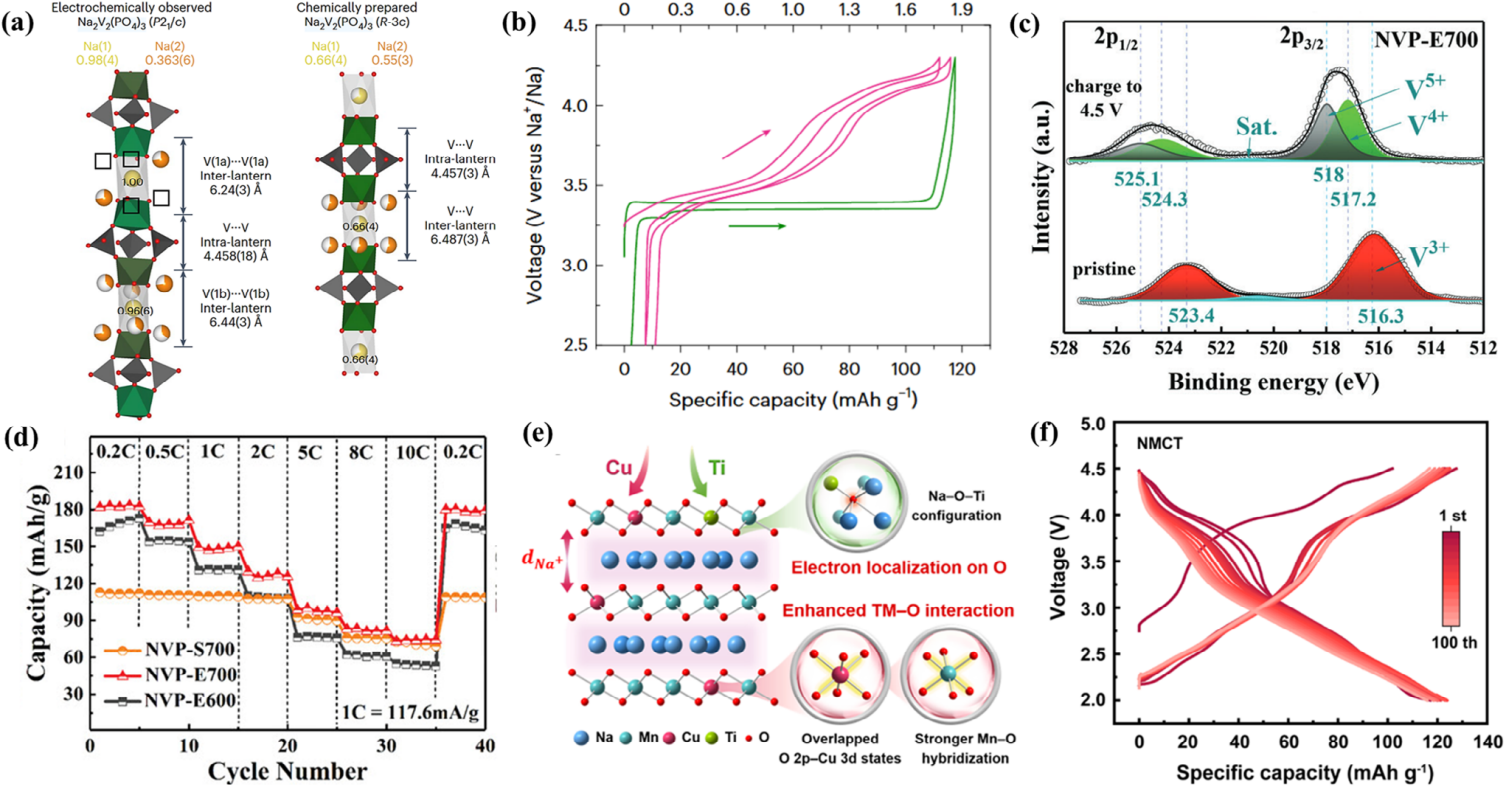
a) Illustrations of the V_2_(PO_4_)_3_ units with neighboring Na^+^ ions in the crystal structures of electrochemically formed e‐Na_2_V_2_(PO_4_)_3_ (left) and chemically prepared c‐ Na_2_V_2_(PO_4_)_3_ (right). b) Charge and discharge curves of Na_2_V_2_(PO_4_)_3_ (green) and c‐ Na_2_V_2_(PO_4_)_3_ (pink) electrodes cycled between 2.5 and 4.3 V at 10 C (one Na^+^ per 10 h) versus Na metal. Reproduced with permission.^[^ ^16^ ^]^ Copyright 2024 Springer Nature. c) High‐resolution XPS spectra of V (2p) for the NVP‐E700 sample before and after charged to 4.5 V. d) Rate performance of the NVP‐S700, NVP‐E700, and NVPE600 electrodes. Reproduced under terms of the CC‐BY license.^[^ ^71^ ^]^ Copyright 2022 The Authors. Published by Advanced Science published by Wiley‐VCH GmbH. e) Schematic illustration of the capacity‐stability win–win engineering for SIBs by the strategy of Cu/Ti co‐doping. f) Cycling performance at 0.1 C for NM and NMCT. Reproduced with permission.^[^ ^70^ ^]^ Copyright 2024 Elsevier B.V.

Na_3_V_2_(PO_4_)_3_ has gained attention as a promising cathode material due to its structural stability, high rate performance, and long cycle life. Utilizing the V^4+/3+^ redox pair (3.37 V vs Na^+^/Na), it delivers a theoretical capacity of 117.6 mAh g^−1^ and an energy density of ≈396 Wh kg^−1^. Two Na^+^ ions can be reversibly exchanged via a biphasic mechanism, but extracting the third Na^+^ is challenging due to high migration energy and increased redox potential. Chemically synthesized c‐Na_2_V_2_(PO_4_)_3_, obtained by calcining Na_3_V_2_(PO_4_)_3_ and Na_1_V_2_(PO_4_)_3_, exhibits a distinct Na^+^ distribution with only 0.66 Na(1) site occupancy, unlike electrochemically synthesized e‐Na_2_V_2_(PO_4_)_3_, where Na site occupancy is ≈1.^[^ ^16^ ^]^ The Na distribution in e‐Na_2_V_2_(PO_4_)_3_ by both synthesized methods is demonstrated in **Figure** **9**a. Unlike conventional Na_3_V_2_(PO_4_)_3_, chemically synthesized Na*_x_* V_2_(PO_4_)_3_ has a unique Na^+^ ion de‐embedding/intercalation mechanism, which is capable of achieving continuous voltage changes during Na^+^ de‐embedding/intercalation. They found that the single crystal phase Na_2_V_2_(PO_4_)_3_ reaches an average potential of ≈3.7 V versus Na^+^/Na during Na^+^ de‐embedding, which is an enhancement of 0.33 V compared to the average potential of Na^+^/Na de‐embedding of conventional Na_2_V_2_(PO_4_)_3_ of 3.37 V versus Na^+^/Na (Figure 9b). This variation affects the Na^+^ intercalation mechanism, enabling a continuous voltage change. The single‐crystal phase Na_2_V_2_(PO_4_)_3_ achieves an average potential of ≈3.7 V versus Na^+^/Na, an improvement of 0.33 V over conventional Na_2_V_2_(PO_4_)_3_. This enhancement increases its theoretical energy density from 396.3 to 458.1 Wh kg^−1^. Ma and colleagues developed self‐supported 3D porous Na_3_V_2_(PO_4_)_3_ (NVP) materials using electrostatic spray deposition (ESD) on carbon foam (CF) substrates.^[^ ^71^ ^]^ By adjusting the annealing temperature, they obtained NVP samples with different crystallinity (NVP‐E700 and NVP‐E600, Figure 9c). The disordered structure, containing both nanocrystalline and amorphous phases, activates the V^5+^/V^4+^ redox pairs and enables a three‐electron reaction, increasing sodium storage capacity compared to fully crystallized NVP (NVP‐S700). The NVP‐E700 sample, calcined at 700 °C, supports simultaneous V^4+^/V^3+^ and V^5+^/V^4+^ redox reactions, expanding the battery's voltage window. It delivers an initial discharge capacity of 179.6 mAh g^−1^ with minimal degradation after 200 cycles, maintaining a 99.6% retention rate. At various discharge rates (0.2 C to 10 C), it achieves high capacities, Figure 9d, demonstrating excellent rate performance and cycling stability, making it a strong candidate for high‐performance SIBs.

To address the trade‐off between capacity and stability at high voltage in SIBs, Zhou and colleagues developed a P2‐type Na_0.67_Mn_0.8_Cu_0.15_Ti_0.05_O_2_ (NMCT) cathode material. Traditional layered oxides suffer from phase transitions (P2 to OP4/O2) due to weak TM─O bonds, affecting performance.^[^ ^70^ ^]^ Lowering the charging voltage to 4 V mitigates these transitions but reduces capacity and voltage. NMCT overcomes these limitations by utilizing complete solid‐solution reactions and reversible oxygen oxidation. Ti doping stabilizes the oxygen framework by strengthening Ti─O bonds, while Cu enhances TM‐O hybridization, increasing structural rigidity (Figure 9e). These modifications prevent the undesired P2‐OP4 phase transition, improve Na^+^ transport kinetics, and maintain structural integrity. As a result, NMCT delivers high specific capacity, excellent rate performance, and long cycling stability in the 2–^−^4.5 V range, Figure 9f. It achieves an initial capacity of 118.4 mAh g^−1^ and retains 95.7% capacity after 100 cycles, demonstrating its potential for high‐performance SIBs.^[^ ^70^ ^]^

Chen and co‐workers present the development of Prussian Blue@Graphene Oxide‐Polyvinylpyrrolidone (PB@GO‐PVP) composite materials for SIB cathodes using microwave radiation (MR) and co‐precipitation (CD) methods.^[^ ^108^ ^]^ The MR method involves reacting solutions containing FeSO_4_·7H_2_O, Na_4_[Fe(CN)_6_]·10H_2_O, and PVP in a microwave reactor to promote covalent bonding between PVP and GO, enabling uniform PB anchoring. The CD method entails mixing solutions in a flask with stirring and dropwise add. The PB@GO‐PVP(MR) composite shows superior electrochemical performance. It achieves an initial specific capacity of 165.2 mAh g^−1^ at 1 C and retains 120.1 mAh g^−1^ after 500 cycles. It also exhibits excellent rate capability, with a discharge capacity of 161.1 mAh g^−1^ at 0.2 C and 97 mAh g^−1^ at 10 C. In addition, Wang et al. discuss the preparation of Prussian blue (PB) analogs by a calcination strategy, which successfully activates low‐spin iron (LS‐Fe) to enhance the electrochemical performance of PB in SIBs.^[^ ^109^ ^]^ The high voltage is achieved through a calcination strategy that activates LS‐Fe in PB, inducing a charge transfer‐induced spin transition during the electrochemical reaction. This involves charges transferring from high‐spin iron (HS‐Fe) to LS‐Fe, changing the electron cloud configuration of LS‐Fe and making it more reactive. PB‐325 exhibited superior rate performance, delivering 110 mA g^−1^ at 5 C (84% capacity retention compared to 0.2 C) and 99 mAh g^−1^ at 10 C (76% capacity retention compared to 0.2 C). An advanced layered oxide cathode material Na_0.79_Lix[Li_0.13_Ni_0.20_Mn_0.67_]O_2_@LiF (NaLi‐LNM@LiF) with a superlattice structure was fabricated by Feng.^[^ ^110^ ^]^ Through electrochemical in situ doping and capping, Li^+^ enters into the layered structure and acts as a “pillar” to stabilize the structure, inhibiting the loss of oxygen at high voltage and thus improving the structural stability of the material at high voltage. The doping of Li^+^ and the formation of the LiF capping layer not only stabilized the lamellar structure but also inhibited the dissolution of transition metal ions and lattice mismatch, reducing the side reactions between the cathode material and the electrolyte, thus significantly improving the electrochemical performance. In the voltage range of 1.5^−^4.5 V, NaLi‐LNM@LiF exhibits 170.6 mA h g^−1^ specific capacity and retention of 92.65% after 200 cycles at 0.5 C current density.

Changing the cathode material synthesis process is one of the important to addressing the trade‐off between capacity and stability at high voltage in SIBs. Stability is decreased by the structural deterioration that materials frequently undergo at higher voltages, including phase transitions and oxygen loss. This can be lessened by using a variety of synthesis procedures, including sol–gel, hydrothermal, and co‐precipitation, which increase stability and capacity. Hydrothermal synthesis, for instance, can enable materials like layered oxides to develop more stable and homogeneous crystal structures, which will improve their resistance to high‐voltage cycling. Similar to this, the sol–gel process produces more robust materials that maintain their structure and functionality after several charge–discharge cycles by precisely controlling particle size and distribution.

### 3.4 Interface Engineering and Its Application to High Voltage Cathode Materials

Optimizing the electrolyte is another efficient way to improve electrochemical performance, in addition to changing the electrode materials and preparation techniques. When operating at high voltages, the cathode material of SIBs encounters several problems, including oxygen precipitation, phase transitions and fragmentation of cathode particles, dissolution and migration of TMs, and oxidation of the electrolyte on the high voltage cathode. Improving electrolyte stability by designing electrolyte additives and using solvents with high antioxidant capacity can help improve the performance of high‐voltage SIBs. Additives can build a stable CEI, which improves the electrochemical performance of the battery by forming a protective layer, and additives can improve the electrochemical performance of the battery by forming a stable CEI through self‐decomposition or interaction with the electrode surface.^[^ ^111^ ^]^

#### 3.4.1 Interfacial Interactions between Electrolyte and Cathode Materials and Their Regulation

**Figure 10:**
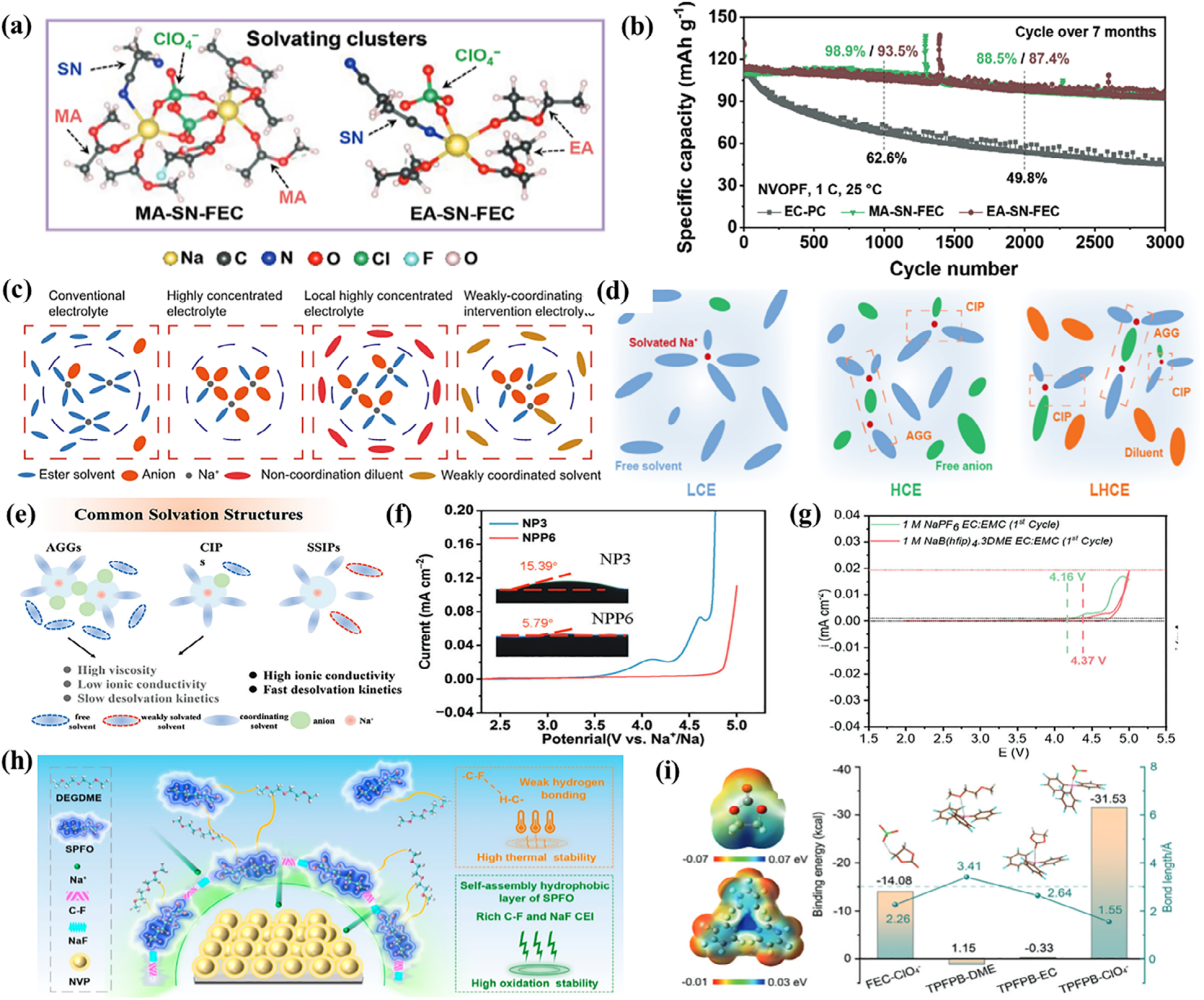
a) Solvating clusters between Na^+^ and solvent ligands in MA‐SN‐FEC and EA‐SN‐FEC electrolytes, observed via molecular dynamics. b) Long‐term cycling performance of the NVOPF half cells at 1 C at 25 °C after three conditioning cycles at 0.1 C. Reproduced with permission.^[^ ^112^ ^]^ Copyright 2024 Wiley‐VCH GmbH. c) Schematic of electrolyte solvation structure from conventional electrolyte to WCIE. Reproduced with permission.^[^ ^113^ ^]^ Copyright 2024 Springer Nature. d) The solvation structure of LCE, HCE, and LHCE. CIP (anion coordinated with one cation), AGG (an anion coordinated with more than one cation. Reproduced with permission.^[^ ^114^ ^]^ Copyright 2024 Elsevier Ltd. e) Illustration of solvation structures highlighting cation environments: AGGs (ion aggregates), CIPs (contact ion pairs), and SSIPs (solvent‐separated ion pairs). f) LSV of NP3 and NPP6 with the inset wetting properties. Reproduced with permission.^[^ ^115^ ^]^ Copyright 2024 Wiley‐VCH GmbH. g) First cycle CV profile to check the oxidative stability of 1 m Na[B(hfip)_4_].3DME EC: EMC and 1 m Na[PF_6_] EC: EMC electrolytes at 0.1 mV s^−1^ on a non‐coated Al current collector. Reproduced with permission.^[^ ^116^ ^]^ Copyright 2024 Wiley‐VCH GmbH. h) Schematic of the ─C─F—H─C─ pseudo‐hydrogen bonding between the additive and the solvent enhances the dispersion and thermal stability. Reproduced with permission.^[^ ^117^ ^]^ Copyright 2024 American Chemical Society. i) Electrostatic potential density distribution of FEC and TPFPB molecule and bond length of FEC‐ClO_4_^−^, TPFPB‐DME, TPFPB‐EC, and TPFPB‐ClO_4_^−^ through H–O, B–O, B–O, and B–O interactions, respectively. Reproduced with permission.^[^ ^118^ ^]^ Copyright 2023 Wiley‐VCH GmbH.

Stabilizing the electrode interface is an urgent requirement for improving the lifetime of high‐voltage SIBs. However, the fragile CEI leads to capacity decay at high voltage, and the solvation‐interphase performance relationship has not been adequately addressed. In order to fill the gap in revealing the solvent‐interfacial property relationship and to further modulate the interfacial components of high‐voltage SIBs, a synergistic Na^+^ solvation strategy was investigated by Liu.^[^ ^112^ ^]^ They used low freezing point, moderately solvated carboxylic acid esters (methyl acetate (MA) or ethyl acetate (EA)) and oxidation‐stabilized succinonitrile (SN) as dual solvents to produce the electrolyte. The electrolyte stabilizes the interfacial phase by combining a soft solvent and a medium co‐solvent in the overall design of the electrolyte, yielding a CEI with ideal thickness and component stability. Representative solvation clusters between Na^+^ and solvent ligands in the electrolyte are shown in **Figure** **10**a. The 4.3 V Na_3_V_2_O_2_(PO_4_)_2_F (NVOPF) cathode with 1 mol sodium perchlorate (NaClO_4_) as the electrolyte and 5% FEC in M: SN (1:1 by volume, M = MA, EA) dual solvents was cycled for 3000 cycles at 1 C, and the capacity retention reached 83.3% significantly better than that of carbonate cathode (41.6%) (Figure 10b). In addition, NVOPF||HC full cells assembled with hard carbon anode electrodes have superior rate capability (up to 15 C) and stable cycling stability of over 500 cycles. Moreover, a cation weakly coordinating‐intervention strategy was proposed by Wang et al.^[^ ^113^ ^]^ Figure 10c presents the schematic design of the electrolyte solvation structure, shifting from a conventional electrolyte to a weakly coordinating‐intervention (WCI)‐based electrolyte (WCIE). The solvation structure of Na^+^ is adjusted by the weak coordination of 1,2‐difluorobenzene (DFBn) with Na^+^ to form a Na^+^‐anion composite structure, which combines with salt decomposition to enhance the formation of the cathode interface. By adjusting the solvation structure of the electrolyte, a 97.5% Coulombic efficiency for 600 cycles at 1 mA cm^−2^ and a beneficial lifetime of 2500 h were achieved for the Na‖Cu cell. Meanwhile, the Na‖PB cell achieves long‐term operation at 4.8 V and can operate over a wide temperature range (−30–70 °C). Furthermore, by dissolving sodium hexafluorophosphate in methyl ethyl carbonate, fluorinated ethylene carbonate, and 1,1,2,2‐tetrafluoroethyl‐2,2,3,3‐tetrafluoropropyl ether, Liu and co‐workers investigated a localization high‐concentration electrolyte (LHCE).^[^ ^114^ ^]^ LHCE named EFT613, which has excellent oxidative and interfacial stability with respect to Na^+^/Na over 6 V. The solvated structure of LHCE is shown in Figure 10d During electrochemical charging at 4.4 V, the preferential decomposition of the PF_6_^−^ anion produces a stable and robust inorganic‐enriched CEI, which reduces the interfacial impedance, facilitates Na^+^ transport, and inhibits the subsequent side reactions. The high voltage P2‐Na_0.7_Li_0.03_Mg_0.03_Ni_0.27_Mn_0.6_Ti_0.07_O_2_ cathode and Na anode cell (Na‐LMNM′T) had a discharge capacity of 129 mA h g^−1^, with a capacity retention of 87.3% after 200 cycles during long‐term cycling at high voltage of 4.4 V. The average CE was 99.8%. Good rate of increase with electrolyte (LHCE), 97.4 mAh g^−1^ at 10 C and 85.6 mAh g^−1^ at 20 C. While high‐concentration electrolytes (HCEs) and the formation of anion‐derived CEI membranes can improve oxidative stability, at the same time high cost, viscosity, and low ionic conductivity remain obstacles in their development. In recent years, the strategy of using WSEs to modulate the Na^+^ solvation structure provides an effective solution for the stability of the high‐voltage cathode electrode/electrolyte interface. WSE not only shows advantages in terms of cost but also takes into account oxidative stability. On this basis, Wang et al. solved the critical bottleneck of electrode/electrolyte interface instability by employing a new ternary weak‐solvent low‐concentration salt electrolyte, (ethoxy(pentafluoro) cyclotriphosphazene (PFPN) through an anion/cation solvation strategy.^[^ ^115^ ^]^ As Figure 10e illustrates the schematic of the anionic/cationic solvation strategy of PFPN, the weakly polarized fluorinated co‐solvent (ethoxy(pentafluoro) cyclotriphosphazene, PFPN) reshapes the intermolecular interactions within the designed electrolyte compared to the conventional carbonate‐based electrolyte. They found that PFPN appeared in the second solvated shell layer of Na^+^, which provided space for solvated Na^+^ migration, reduced the coordination number of Na^+^ with PC and ClO_4_^−^, separated PC from Na^+^, accelerated the dual solvation kinetics of Na^+^, and enhanced the antioxidant stability of the electrolyte. The ionic conductivity of the low‐concentration electrolyte was increased to 6.12 mS cm^−1^ and the oxidative stability was successfully extended to 4.84 V (Figure 10f). Na_3_V_2_(PO_4_)_2_F_3_ (NVPF)‐Na half‐cells exhibit excellent cycling performance, with an average Coulombic efficiency of 99.5% after 2000 cycles at 4.5 V. The NVPF//HC full‐cells have relatively high energy density (≈450 Wh kg^−1^).

In addition to solvation strategies, additives can improve the electrochemical performance of batteries by forming stable solid CEIs through self‐decomposition or interaction with the electrode surface. Lohani and co‐workers added high‐fluoroborate anions to a conventional electrolyte (1 m Na[PF_6_] EC: EMC) made of (1 m Na[B(hfip)_4_]. 3DME) a novel electrolyte composition with enhanced performance in high voltage sodium all‐cell batteries.^[^ ^116^ ^]^ As shown in Figure 10g, the stability of 1 m Na[B(hfip)_4_].3DME EC: EMC electrolyte is as high as 4.35 V (vs Na/Na^+^), which exceeds the highest operating voltage (4.2 V) of NVPF@C@CNT and NFMO cathodes, whereas the stability of 1 m Na[PF_6_] EC: EMC electrolyte is only 4.16 V. They found that the improved electrochemical stability was due to the formation of sodium fluoride (NaF)‐rich and boron‐containing (BO and B‐F) CEIs on the NVPF@C@CNT and NFMO cathode surfaces by the 3DME EC: EMC electrolyte. These NaF and boron‐containing components inhibited electrolyte degradation at high voltages, thus improving the cycling performance of full sodium batteries. They found that NVPF@C@CNT/Na full batteries using 1 m Na[B(hfip)_4_].3DME EC: EMC electrolyte had a capacity retention of 93.3% after 100 cycles at a current density of 0.1 C. In contrast, batteries using 1 m Na[PF_6_] EC: EMC electrolyte under the same conditions had a capacity retention of only 30%. Similarly, Hou et al. proposed a self‐assembled protective layer using perfluorinated anionic additive (SPFO) as a biphasic electrolyte surfactant and simultaneously constructed ─C─F···H─C─ stabilized interaction network.^[^ ^117^ ^]^ As demonstrated in Figure 10h, the ─C─F···H─C─ pseudo hydrogen bonding between the additive and the solvent enhances the dispersion and thermal stability of the material, which allows the cycling performance of the Na_3_V_2_(PO_4_)_3_(NVP)‐based cathode material in SPFO‐containing electrolyte at 60 °C was dramatically improved. The capacity retention was 83%, 90.6%, and 95% for 1 C 50, 5 C 100, and 10 C 100 cycles, respectively, when the rate test was performed at 60 °C. In addition, the use of SPFO increases the stability of ether‐based electrolytes based on Na_3_V_2_(PO_4_)_3_(NVP) SIBs, thereby extending the operating voltage. The preferential adsorption and oxidation of the additives in the first place causes the electrolyte to inhibit weak oxidation at low voltages, which results in the formation of a protective CEI that contributes to the high oxidation resistance and fast kinetics of the electrolyte. In a further step, the SPFO can withstand high voltages of up to 4.5 V versus Na/Na^+^, which promotes the uniform deposition of Na and inhibits the growth of Na dendrites. In addition, the additives work to improve the ability of the battery to adapt to seasonal and daily variations in operating temperature. Generally, the decomposition of the electrolyte on the cathode surface intensifies quickly at high temperatures. The formed CEI tends to be unstable, with continuous electrolyte decomposition, surface reconstruction, and capacity degradation often occurring. The operation of SIBs at high voltages and high temperatures further exacerbates the defects of the unstable and uncontrollable CEI, and oxidative stability becomes significantly narrower. In order to satisfy the requirements of high‐voltage and high‐temperature SIBs and to carefully construct robust and stable CEIs, Zhou proposed the use of the anionic acceptor tris(pentafluorophenyl)‐borane (TPFPB), featuring electron‐deficient boron (B) centers, to control solvation conformations (Figure 10i).^[^ ^118^ ^]^ This anion weakens ClO_4_^−^ solvation through strong interactions between electron‐deficient boron atoms and TPFPB facilitates the coordination capacity between the solvent and the Na^+^ cation, and greatly improves oxidative stability. In a working NVP cathode with a high cutoff voltage of 4.2 V (vs Na^+^/Na), the electrolyte EDT with the addition of 3 wt.% TPFPB provided 100 cycles at 60 °C with a capacity retention of 86.9%, whereas the capacity retention of the electrolyte ED with unadditived electrolyte (1 m NaClO_4_‐EC/DME) electrolyte was only 66.6%. Not only does the type of additive have an effect on the electrochemical performance of the battery, but the concentration of the additive also plays a crucial role in enhancing the electrochemical performance of the battery material. Jayakumar used FEC as a typical film‐forming additive at different concentrations (2, 5, and 10 wt.%) and demonstrated that in 210 mAh g^−1^ Na_0.97_Ca_0.03_[Mn_0.39_Fe_0.31_Ni_0.22_Zn_0.08_]O_2_ (NCMFNZO)/HC pouch cells, ethyl acetate (EA) as the only feasibility of weak solvents.^[^ ^119^ ^]^ FEC additives can improve the high‐voltage performance of batteries because the CEI layer formed by FEC can effectively protect the electrode materials from structural damage and over‐oxidation at high voltages. They found that a 5 wt.% FEC concentration improves battery cycle life and capacity retention while also maintaining a low charge transfer resistance, improving the multiplicative performance of the battery and enabling it to better maintain capacity and life during rapid charging. The NCMFNZO/HC battery achieves a long cycle life of 250 cycles, cycling between 1.5 and 4 V and maintaining 80% capacity at 40 C. The electrochemical performance results of SIBs in full‐cell mode with NASICON cathode and various other materials as an anode are listed in **Table** **3**.

**Table 3:** Overview of electrolyte compatibility, voltage ranges, and electrochemical performance of full cell for SIBs.

Anode	Cathode	Electrolyte	Working voltage [V]	Rate capacity	Cycling stability	Refs.
HC	Na_3_V_2_O_2_(PO_4_)_2_F (NVOPF)	1 m NaClO_4_ in M/SN (1:1 v/v, M = MA, EA) +5%FEC	2–4.3		Retained 83.3% after 3000 cycles at 1 C	[112]
Na	Prussian Blue (PB)	1.7 m NaTFSI in TMP‐FEC‐1,2‐DFBn (2: 1: 5 w/w)	2.4–4.5	98.1 mA h g^−1^ at 10 C	Retained 85% after 150 cycles at 10 C	[113]
Na	P2‐Na_0.7_Li_0.03_Mg_0.03_Ni_0.27_Mn_0.6_Ti_0.07_O_2_(Na‐LMNM′T)	1 m NaPF_6_ in EMC/FEC/TTE(6:1:3 v/v)	2.2–4.4	84 mA h g^−1^ at 20 C	Retained 87.3% after 200 cycles at 1 C	[114]
HC	Na_3_V_2_(PO_4_)_2_F_3_ (NVPF)	1 m NaClO_4_ in PC/PFPN (7.5:1 v/v)	2–4.5	114.6 mA h g^−1^ at 50 mA g^−1^	Retained 84.2% after 300 cycles at 1 C	[115]
Na	NVPF@C@CNT	1 m Na[B(hfip)_4_].3DME EC/EMC (3:7 v/v)	1.5–4.5	123.9 mA h g^−1^ at 0.1 C	Retained 93.3% after 100 cycles at 0.1 C	[116]
Na	Na_3_V_2_(PO_4_)_3_ (NVP)	0.1 m NaPF_6_ in DEGDME/DOL (10:1 v/v)	1.5–4.5	115 mA h g^−1^ at 0.1 C	Retained 93.3% after 100 cycles at 0.1 C	[117]
Na	NVP	1 m NaClO_4_‐EC/DME (1:1 v/v) + 3 wt.% TPFPB	– 4.2	115 mA h g⁻^1^ at 0.25 C	Retained 86.9% after 100 cycles at 0.5 C	[118]
HC	Na_0.97_Ca_0.03_[Mn_0.39_Fe_0.31_Ni_0.22_Zn_0.08_]O_2_	1 m NaPF_6_ in EC + 5 wt.% FEC	1.5–4		Retained 80% after 250 cycles at 440 C	[119]
Na	NaNi_1/3_Mn_1/3_Fe_1/3_O_2_	1.2 m NaTFSI in SUL/OTE (1:1) + 5 wt.% FEC	2–4.2	130.82 mA h g⁻^1^ at 1 C	Retained 81.15% after 400 cycles at 2 C	[66]

The electrolyte has a major impact on the safety and performance of high‐voltage SIBs. Carbonate‐based electrolytes are widely used because of their strong ionic conductivity; nevertheless, they experience oxidative breakdown at high voltages, which can lead to gas evolution, electrolyte depletion, and potentially thermal runaway, particularly in layered oxide cathodes. This degradation not only reduces battery life but also poses safety issues. Conversely, ionic liquid electrolytes reduce flammability and avoid high‐voltage breakdown by offering superior thermal and electrochemical stability. However, because of their higher viscosity, ion mobility is decreased, which limits rate capability and overall battery performance. The cost of ionic liquids and their challenging manufacturing process further hinder their widespread use.

Full form for the various abbreviations used in the table is given: EC‐ Ethylene Carbonate, PC‐ propylene carbonate, FEC‐ Fluoroethylene Carbonate, DMC‐ Dimethyl Carbonate; DME‐ Dimethoxy Ethane; NaTFSI‐ Sodium bis (trifluoromethanesulfonyl) imide; SUL‐ Sulfolane (a sulfone‐based solvent); OTE‐ 1H, 1H, 5H‐octafluoropentyl‐1, 1, 2, 2‐tetrafluoroethyl ether (a non‐solvent diluent); DEGDME‐ Diethylene Glycol Dimethyl Ether; MA‐methyl acetate; EA‐ ethyl acetate; SN‐ oxidation‐stabilized succinonitrile; PFPN‐ (ethoxy(pentafluoro) cyclotriphosphazene; EMC‐ethyl methyl carbonate; TPFPB‐tris (pentafluorophenyl)‐borane.

#### 3.4.2 CEI

Formed on the battery's cathode electrode surface, the CEI is a composite film made up of electrolyte decomposition products. These products exhibit ionic conductivity and electronic insulation properties. The CEI prevents direct contact between the cathode material and the electrolyte, reduces side reactions, and thus improves battery performance. The CEI layer of conventional cathode materials usually consists of organic and inorganic components, where inorganic components such as NaF, Na_2_O, Na_2_CO_3_, etc., and organic components may include ROCO_2_Na, RONa, etc., whereas the CEI of high‐voltage cathode materials may require more inorganic components to improve its stability at high voltages in order to prevent the continuous decomposition of the electrolyte and the degradation of the battery performance.^[^ ^120^ ^]^ For conventional cathode materials, the performance of CEI can be optimized by means of electrolyte additives, current density, temperature, and electrode surface modification. In the case of high‐voltage cathode materials, in addition to the methods previously discussed, it is crucial to develop new electrolyte additives and interface modulation strategies to adapt to higher voltage requirements and boost the battery's cycling stability.

**Figure 11:**
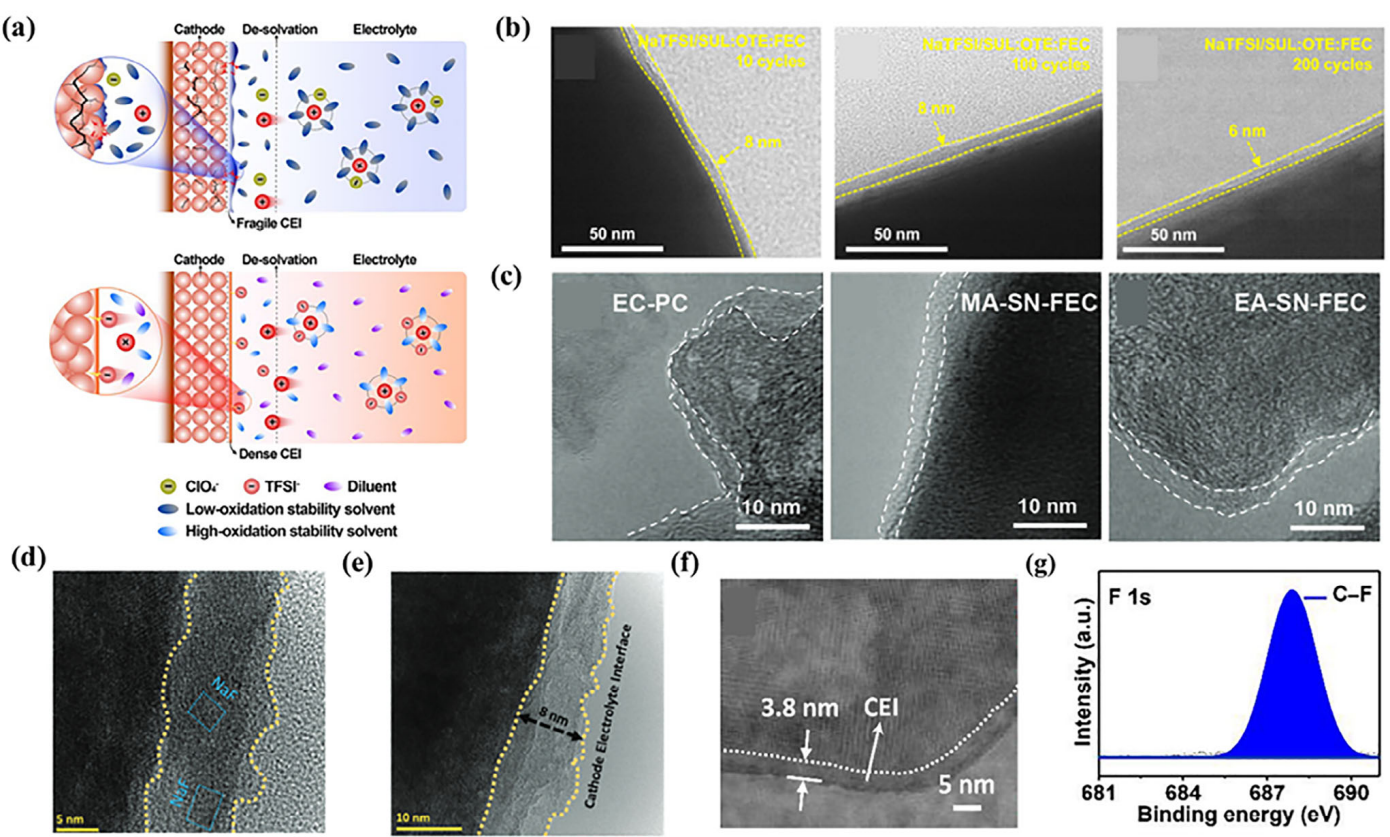
a) Schematic illustrations of the solvation structure and the CEIs in the E‐Control and NaTFSI/SUL:OTE: FEC. b) TEM images of NaNMF cathode cycled in the NaTFSI/SUL:OTE: FEC. Reproduced under terms of the CC‐BY license.^[^ ^66^ ^]^ Copyright 2024, The Authors, published by Springer Nature. c) HR‐TEM images for the EC‐PC, MA‐SN‐FEC, and EA‐SN‐FEC electrolytes derived CEI, as schematically shown by the white dashed lines. Reproduced with permission.^[^ ^112^ ^]^ Copyright 2024 Wiley‐VCH GmbH. d) HR‐TEM image showing the formation of inorganic crystallites (NaF) on the surface of the NFMO cathode present in the CEI. e) TEM and HR‐TEM images showing CEI of 8 nm thickness formed on the surface of the NFMO cathode after 100 cycles. Reproduced with permission.^[^ ^116^ ^]^ Copyright 2024 Wiley‐VCH GmbH. f) TEM images of CEI@PB before cycling. g) High‐resolution XPS profiles of the PB cathode in the C 1s and F 1s regions. Reproduced with permission.^[^ ^121^ ^]^ Copyright 2021 Elsevier Ltd.

Conventional carbonate electrolytes have low oxidative stability and poor passivation at high voltages, making it difficult to form a stable CEI above 4 V (vs Na^+^/Na). To address this, researchers have designed electrolytes to create stable CEIs. For example, He et al. introduced a highly antioxidant sulfoxide solvent combined with a non‐solvent diluent, 1H,1H,5H‐octafluoropentyl‐1,1,2,2‐tetrafluoroethyl ether, in high‐concentration electrolytes. This electrolyte demonstrated excellent oxidative stability and formed a thin, dense, and homogeneous CEI (**Figure** **11**a).^[^ ^66^ ^]^ The stable CEI enabled efficient cycling of the O3‐type NaNi_1/3_Mn_1/3_Fe_1/3_O_2_ cathode. The NaTFSI/SUL:OTE: FEC electrolyte showed capacity retention of 79.48% after 300 cycles at 1 C and 81.15% after 400 cycles at 2 C with a 4.2 V charging voltage. The non‐flammability of the electrolyte also improved the safety of SIBs. The thin and dense CEI effectively reduced side reactions and minimized volume changes, as seen in the structural characterization (Figure 11b). It isolated the cathode from continuous reactions with the electrolyte, preventing further surface cracks and improving the overall stability and performance of the battery. Similarly, Liu and co‐workers obtained stable CEIs by designing cosolvent electrolytes containing methyl carboxylates (e.g., methyl acetate MA and ethyl acetate EA) and butanedinitrile (SN).^[^ ^112^ ^]^ These cosolvent electrolytes were rationally adjusted to the Na^+^ solvation environment to produce F/N inorganic‐rich (e.g., NaF and Na_3_N) as well as ideally thick and component‐stable CEIs (Figure 11c), which resulted in a high degree of compatibility with high‐voltage NVOPF cathodes and HC anodes. In a 4.3 V Na_3_V_2_O_2_(PO_4_)_2_F (NVOPF) cathode, a capacity retention of 83.3% over 3000 cycles was achieved using this design of electrolyte, which is significantly better than that of conventional carbonate‐based electrolytes (41.6% capacity retention). In addition, the NVOPF / HC full cell is stabilized for over 500 cycles at 1 C under simultaneously regulated SEI and CEI.

In addition, researchers have used increasing the number of inorganic compounds in the CEI components to enhance CEI. Lohani demonstrates the enhanced performance in high‐voltage sodium full cells using a novel electrolyte composition featuring a highly fluorinated borate ester anion (1 m Na[B(hfip)_4_].3DME) in a binary carbonate mixture (EC: EMC).^[^ ^116^ ^]^ The 3DME EC: EMC electrolyte forms NaF‐rich and boron‐containing (BO and B‐F) CEIs on the surface of the NVPF@C@CNT and NFMO cathodes. These NaF^−^ and boron‐containing constituents inhibit the degradation of the electrolyte at high voltages, thereby improving the cycling time of the all‐sodium battery. The structure and composition of the CEI after prolonged cycling were analyzed using TEM and XPS, revealing 5 and 8 nm thick NaF‐rich inorganic crystals in the HR‐TEM images of NVPF@C@CNT and NFMO cathodes, respectively (Figure 11d,e).^[^ ^116^ ^]^ Similarly, Ye proposed a chemical pre‐naturation strategy to create a NaF‐rich CEI in situ on a PB cathode, improving the cycle life and performance of SIBs.^[^ ^121^ ^]^ A redox reaction between metallic sodium and PVDF binder formed a uniform, dense NaF‐rich CEI, ≈3.8 nm thick, on the PB surface, resulting in a CEI@PB structure (Figure 11f). This CEI consists of inorganic Na salts (NaF, Na_2_CO_3_, and NaHCO_3_) and organic Na‐based species (R‐O‐Na, R‐CO‐Na, R‐O_2_CO‐Na) (Figure 11g). The NaF‐rich CEI provides excellent electronic insulation and ionic conductivity, enabling rapid Na^+^ ion transport and maintaining a stable voltage plateau during cycling. Thanks to this NaF‐rich CEI, CEI@PB shows excellent cycling stability, achieving an area capacity of 0.61 mAh cm^−2^ and lasting 3000 cycles at 1  C.

The development of a CEI is essential in liquid electrolyte systems because it passivates the cathode surface while still allowing Na^+^ transport. To improve stability and lessen undesirable side reactions, this interphase can be improved using electrolyte engineering, additives, and surface coatings. It is worth mentioning that solid‐state batteries have particular difficulties because of solid–solid contact constraints, resulting in interfacial gaps and high resistance, which hinder ion transport, in contrast to liquid electrolytes that guarantee uniform contact. Furthermore, unfavorable side reactions brought on by chemical instability at the interface might result in resistive layers that impair performance.

## 4 Practical Applications of High‐Voltage Cathode Materials

### 4.1 Safety Studies in SIBs

The safety of high‐voltage cathode materials for SIBs during overcharging and over‐discharging is directly tied to the overall safety and longevity of the batteries. Additionally, the thermal stability of cathode materials is a critical element in battery safety. Studies have demonstrated that the onset temperature of the reaction between cathode materials and electrolytes increases with a reduction in the stoichiometric number. Thus, safety research can help to further improve the technical maturity and economic applicability of SIBs, and promote their commercialization in electric vehicles, energy storage, and other fields. In this regard, we summarize the performance of high‐voltage cathode materials in terms of overcharge, over‐discharge, and thermal stability.

#### 4.1.1 Performance of High‐Voltage Cathode Materials under Overcharging

**Figure 12:**
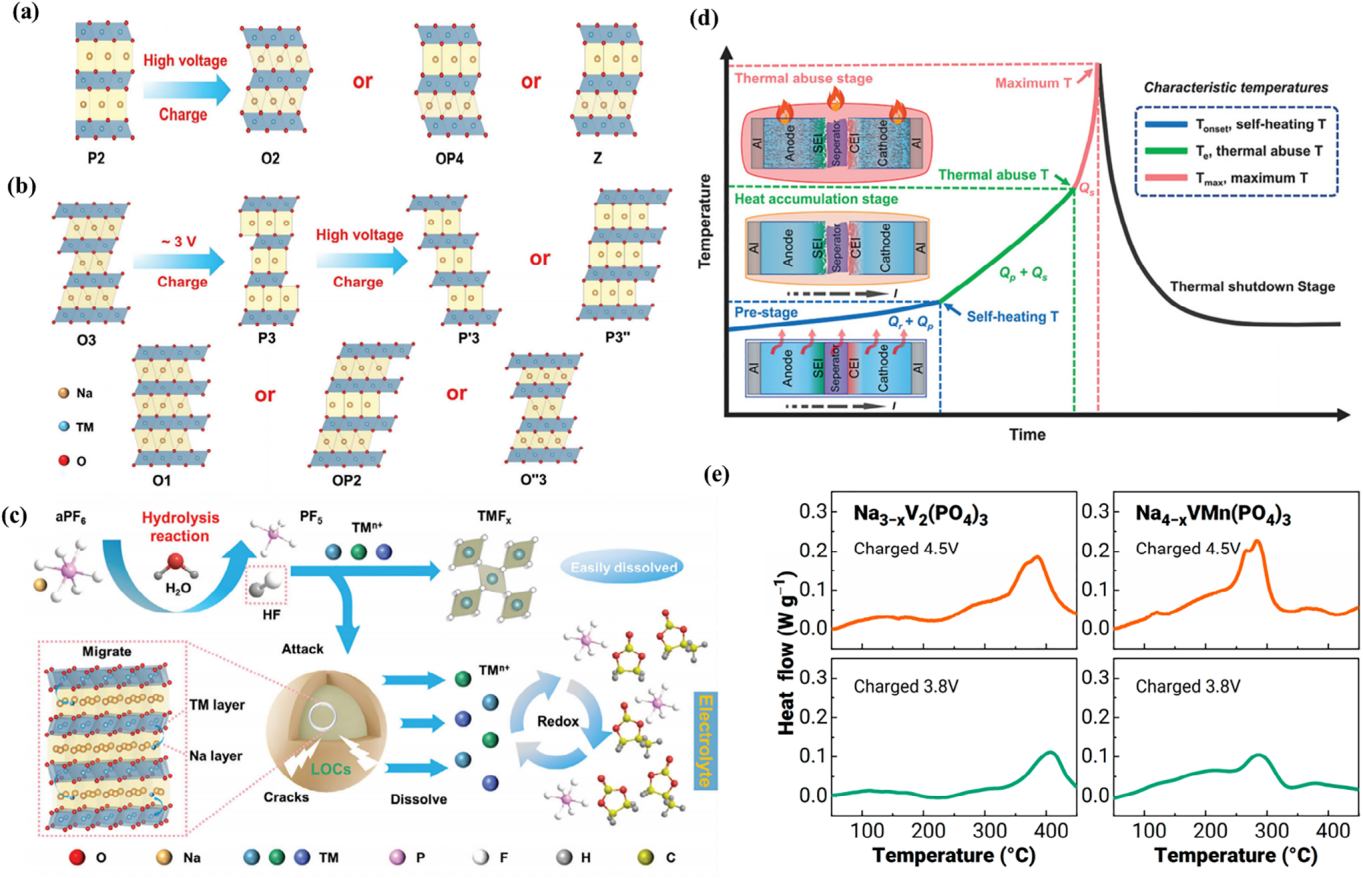
A schematic diagram of the irreversible phase transitions of a) P2 and b) O3 typed LOCs. c) Schematic illustration of TM ion migration and dissolution in LOCs. Reproduced with permission.^[^ ^47^ ^]^ Copyright 2024 Wiley‐VCH GmbH. d) Schematic diagram of the stages and overall process of thermal runaway in SIBs. Reproduced with permission.^[^ ^125^ ^]^ Copyright 2024 Elsevier B.V. e) DSC curves for the charged electrodes containing Na_3_V_2_(PO_4_)_3_ and Na_4_VMn(PO_4_)_3_ cathode materials at different potential cut‐offs (3.8 V on the bottom and 4.5 Von the top). Reproduced with permission.^[^ ^136^ ^]^ Copyright 2023 Licensee MDPI, Basel, Switzerland.

Overcharging refers to the excessive embedding of Na^+^ in the cathode material during battery charging, which may lead to a series of problems within the battery such as uncontrolled chemical reactions in the absence of reactive oxygen species; structural phase transitions; decomposition of the battery's CEI, and thermal runaway. This section focuses on the structural failure of high‐voltage cathode materials under overcharging. In general, P2‐type layered oxide cathode materials experience significant structural phase transitions, volume shrinkage, and particle rupture at battery charging voltages higher than 4.1 V. Specifically, this high‐voltage charging leads to a significant extraction of sodium ions from the cathode lattice of the layered oxide cathode, thereby weakening the shielding between the oxygen layers. As the Coulomb repulsion between the oxygen layers increases, sliding of the TMO_2_ plate is triggered. This sliding not only destroys the bulk structure of the material but may also lead to particle rupture. Along with the sliding of the TM layer, the P2‐type layered oxide cathode transforms from the P2 phase to the O2 phase and is accompanied by a volume shrinkage of ≈20%. This volume change reduces the stability of the structure, which in turn leads to capacity degradation of the battery (shown in **Figure** **12**a). In addition, as shown in Figure 12b, the O3 phase undergoes more complex phase transitions during the extraction of Na^+^ ions at high charging voltages compared to the P2 phase transition, and these changes lead to an increase in lattice distortions, which in turn leads to significant degradation of the cell performance.^[^ ^122^ ^]^ The O3 phase's complex phase transition, which contrasts with the phase transition mechanism of P2‐type layered oxides, is mainly due to the sliding of the TM layer, the Jahn–Teller effect, and fluctuations in the TM─O bond strength. For example, Komaba and others found that O3‐NaNi_0.5_Mn_0.5_O_2_ undergoes a series of phase transitions from O3 to O″3, P3, P″3, and P3″ upon charging to 4.5 V. O3‐NaNi_0.5_Mn_0.5_O_2_ was also found to transition through these phases.^[^ ^123^ ^]^ The different sliding modes of the TM layer during these phase transitions contributed to the formation of new O phases, leading to a significant decrease in d spacing and unit cell volume. These successive phase transitions not only increased the overpotential and voltage polarization but also adversely affected the cycle life of the cells. Therefore, an in‐depth understanding and control of these phase transition processes are essential to enhance the performance and stability of SIBs.

At higher charge cutoff voltages, metal cations (e.g., Co, Cr, and Fe ions) undergo migration from the TM layer to the sodium layer (Figure 12c), especially metals such as Ni and Mn, which are more soluble than other metals due to the manifestation of the Jahn–Teller effect. It has been demonstrated that divalent manganese ions (Mn^2+^) are capable of reacting with the by‐product HF in the electrolyte to form soluble MnF_2_. The solvation of this metal ion leads to the degradation of the TMO_2_ layer, causing a lattice mismatch that ultimately leads to the formation of cracks and noticeable volume changes in the layered oxide cathode material. Under high‐voltage charging conditions, the migration of this metal cation can have a number of detrimental effects on battery performance, including voltage hysteresis, irreversible anion redox reactions, and reduced cycling stability.^[^ ^47^ ^]^ Gui and co‐workers conducted overcharge thermal runaway experiments on a NaNi_1/3_Fe_1/3_Mn_1/3_O_2_||HC pouch battery with a capacity of 3.27 Ah.^[^ ^124^ ^]^ Experimental results demonstrate that overcharging amplifies side reactions, gas production, and sodium metal deposition, leading to more intense exothermic reactions. As overcharging continues, side reactions become more severe, heat accumulates, and the battery temperature gradually rises, which may induce thermal runaway, marked by a rapid temperature increase and a potential risk of fire or explosion.^[^ ^125^ ^]^ In addition, the internal resistance of the battery including ohmic impedance, CEI layer impedance, charge transfer impedance, and Warburg impedance increased after the overcharge cycle.^[^ ^126^ ^]^ Conventional carbonate‐based electrolytes undergo oxidative decomposition at a voltage of ≈4.2 V leading to electrolyte failure and release of gases (e.g., CO_2_ and HF), which may reduce ionic conductivity and affect the charging and discharging performance of the battery. When this voltage is exceeded, oxidation of the electrolyte at the CEI is accelerated, leading to electrolyte decomposition and excessive growth of the CEI layer, which affects the cycling stability of SIBs.^[^ ^7^ ^]^ In addition, electrolytes under overcharging face safety issues, Santamaria et al. found that conventional alkyl carbonates burned violently under flame in flammability tests, while ionic liquid electrolytes were virtually nonflammable, thus pyrrolidine‐based ionic liquid electrolysis for the development of safe SIBs for the incompatibility of conventional electrolytes under high voltage showed promising results.^[^ ^127^ ^]^

In summary, overcharging not only causes structural damage to the cathode material of the battery but also may lead to serious safety issues, including thermal runaway and drastic degradation of battery performance. Therefore, in‐depth study and effective control of the overcharging behavior of SIBs are crucial to ensure the safety and extend the service life of the batteries.

#### 4.1.2 Effects of Over‐Discharge on High‐Voltage Cathode Materials

Over‐discharge refers to the excessive de‐embedding of sodium ions from the cathode material during the discharge process of an SIB, a process that may cause damage to the internal structure of the battery, which in turn affects the performance and service life of the battery. The excessive de‐embedding of sodium ions during cycling at low potentials may lead to irreversible changes in the structure of the cathode material. Especially for layered oxide cathode materials, over‐discharge may trigger complex phase transitions, which not only cause mechanical degradation but also hinder the diffusion of Na^+^, leading to the instability of the electrode–electrolyte interface and the deterioration of the electrochemical performance. In addition, the cathode material after over‐discharge is particularly sensitive to the environment, and once exposed to air, its hygroscopicity will lead to sodium ions in the lattice to be dislodged from the electrode surface, hindering the reversible embedding/dislodging of sodium ions, which will lead to a serious drop in the capacity of the battery. Although the effect of over‐discharge on cycling performance is small compared to overcharging, the rate of decline is relatively slow.^[^ ^128^ ^]^ Under over‐discharge conditions, the cyclability of the battery is similarly affected. Studies have shown that when the discharge voltage drops below 0.2 V and after 100 cycles, the increment in the internal resistance of the over‐discharged battery is insignificant, only 0.9%. However, as the number of cycling weeks increased, the internal resistance increased significantly, to 60.4% of the initial internal resistance by 500 cycles.^[^ ^129^ ^]^ Moreover, over‐discharge may cause safety issues. During the over‐discharge process, metal may be deposited on the surface of the cathode electrode material to form dendrites, which can lead to CEI film decomposition, damage to the pole piece, and the risk of micro‐short circuits between diaphragm pores. These short‐circuits may be amplified in actual use, leading to serious safety issues.

Although the impact of over‐discharge on battery performance is not as drastic as over‐charging, its long‐term effects cannot be ignored, especially in terms of safety performance. Therefore, reasonable control of the discharge depth of the battery to avoid over‐discharge is essential to maintain the stability and safety of the battery.

#### 4.1.3 Thermal Stability Performance of High‐Voltage Cathode Materials

The thermal runaway process of SIBs can be divided into four stages: pre‐stage, thermal accumulation stage, thermal runaway stage, and thermal shutdown stage, as shown in Figure 12d, which demonstrates the schematic diagram of the thermal runaway stage of SIB. This process involves three critical temperature nodes, which are the self‐heating temperature (Tonset or T1), the thermal abuse temperature (Te or T2), and the maximum temperature (Tmax or T3).^[^ ^125^ ^]^ The initial phase usually occurs when the SIB ages naturally, is subjected to external shocks, or is overcharged or discharged, when the battery begins to accumulate heat. The start‐up temperature is generally considered to be Tonset when the rate of temperature increase due to heat release reaches 0.02 °C min^−1^.^[^ ^130^, ^131^, ^132^ ^]^ Afterward, as the temperature gradually increases, the cell enters the heat buildup phase. During this phase, due to the decomposition of the CEI layer, the cell begins to release gas and generate heat, further heating the cell. As the temperature of the cell increases, the CEI decomposition intensifies, triggering a continuous reaction and temperature rise. The thermal abuse temperature, or Te, is reached when the battery's self‐heating rate hits 1 or 10 °C s^−1^, signaling the start of the thermal runaway phase.^[^ ^133^ ^]^ The battery goes through a number of intense reactions during this phase, producing a lot of gas and heat as well as a sharp increase in internal voltage and temperature. Finally, as the cell ruptures and the material is expelled, the battery enters the thermal shutdown phase, where heat is dissipated to the surroundings, and the temperature rapidly decreases. When the battery's reactants run out, the thermal runaway process is over. Negative chain reactions inside the battery are the source of thermal runaway, which can result in dangerous incidents like battery fires and explosions by producing excessive heat and the emission of toxic gasses.

For SIBs, the thermal stability of cathode materials like transition metal oxides, Prussian blue analogs, and polyanionic compounds is essential for ensuring safety. Battery safety is directly impacted by the structural stability and oxygen release of these materials at high temperatures. For example, at high temperatures, layered oxides release oxygen and go through phase changes.^[^ ^134^ ^]^ This oxygen may react with the electrolyte to produce heat, which might result in an explosion, fire, or thermal runaway. At high temperatures, PBAs breakdown their structure by releasing cyanide gas and water.^[^ ^135^ ^]^ Samigullin et al. discovered that the exothermic danger of polyanionic compounds increases with increasing voltage.^[^ ^136^ ^]^ For instance, Na_3_V_2_(PO_4_)_3_ exhibits an exothermic peak at 415 °C and a 78 J g^−1^ heat release at 3.8 V. In comparison to the 3.8 V charge, the start temperature decreases to 337 °C and the exothermic impact rises to 151 J g^−1^, almost doubling the heat release.

Similarly, Na_4_VMn(PO_4_)_3_ exhibits an exothermic peak at 287 °C with a total enthalpy change of 163 J g^−1^ in the 3.8 V charging state, and when the charging voltage is increased to 4.5 V, the intensity of the exothermic peak increases and the total enthalpy change increases to 239 J g^−1^ (Figure 12e). These data show that increasing the charging voltage leads to an increase in the exothermic effect for both materials, especially for the Na_3_V_2_(PO_4_)_3_ material, which shows a significant increase in the exothermic effect at the 4.5 V charging state. This could be attributed to the augmented side reactions between the electrode material and the electrolyte in the high‐voltage charging state, causing more heat to be released. By changing the bonding structure, doping many elements in the TM layer, and replacing or doping particular elements in the cathode structure, the researchers used a high‐entropy approach to improve structural stability and electrochemical performance. These modifications improve thermal safety, prevent oxygen leaks, and encourage phase transitions during riding.

The thermal runaway trigger temperature of SIB usually higher than that of LIBs, and the decomposition temperatures of sodium cathode materials currently used commercially, such as NaNi_1/3_Fe_1/3_Mn_1/3_O_2_ and Na_4_Fe_2_(PO_4_)_2_(P_2_O_7_), are usually higher than those of materials such as nickel–cobalt–manganese oxide (NMC) used in LIBs. This means that under the same operating conditions, SIBs are less prone to thermal runaway when experiencing extreme conditions such as overcharging and short‐circuiting.^[^ ^134^, ^137^ ^]^ In addition, studies have shown that release relatively fewer types and amounts of gases during thermal runaway, and the gases generated by their reactions (e.g., CO_2_ and H_2_) are less reactive at high temperatures. This results in fewer flammable gases being produced in SIBs when thermal runaway occurs, thus reducing the risk of fire and explosion.^[^ ^130^ ^]^

### 4.2 Analysis of Material Degradation Mechanisms and Failure Modes

When operated in high‐voltage environments, cathode materials for SIBs may encounter a number of challenges, including irreversible phase transitions, cation migration, transition metal dissolution, interfacial reactions, and loss of reactive oxygen species. These problems can be broadly categorized into two main groups: bulk phase degradation and interfacial degradation. Through in‐depth analysis of the degradation mechanisms and failure modes of high‐voltage cathode materials for SIBs, the material design can be targeted and optimized to remain stable at high voltages. Such optimization not only helps to develop SIBs with both high energy density and stable cycling performance but also effectively prevents battery failure and reduces the risk of safety accidents, thus significantly improving the overall safety of the battery. Through these efforts, the application prospects of SIBs will be broader, bringing more innovations and breakthroughs in the field of energy storage.

#### 4.2.1 Mechanical Degradation

**Figure 13:**
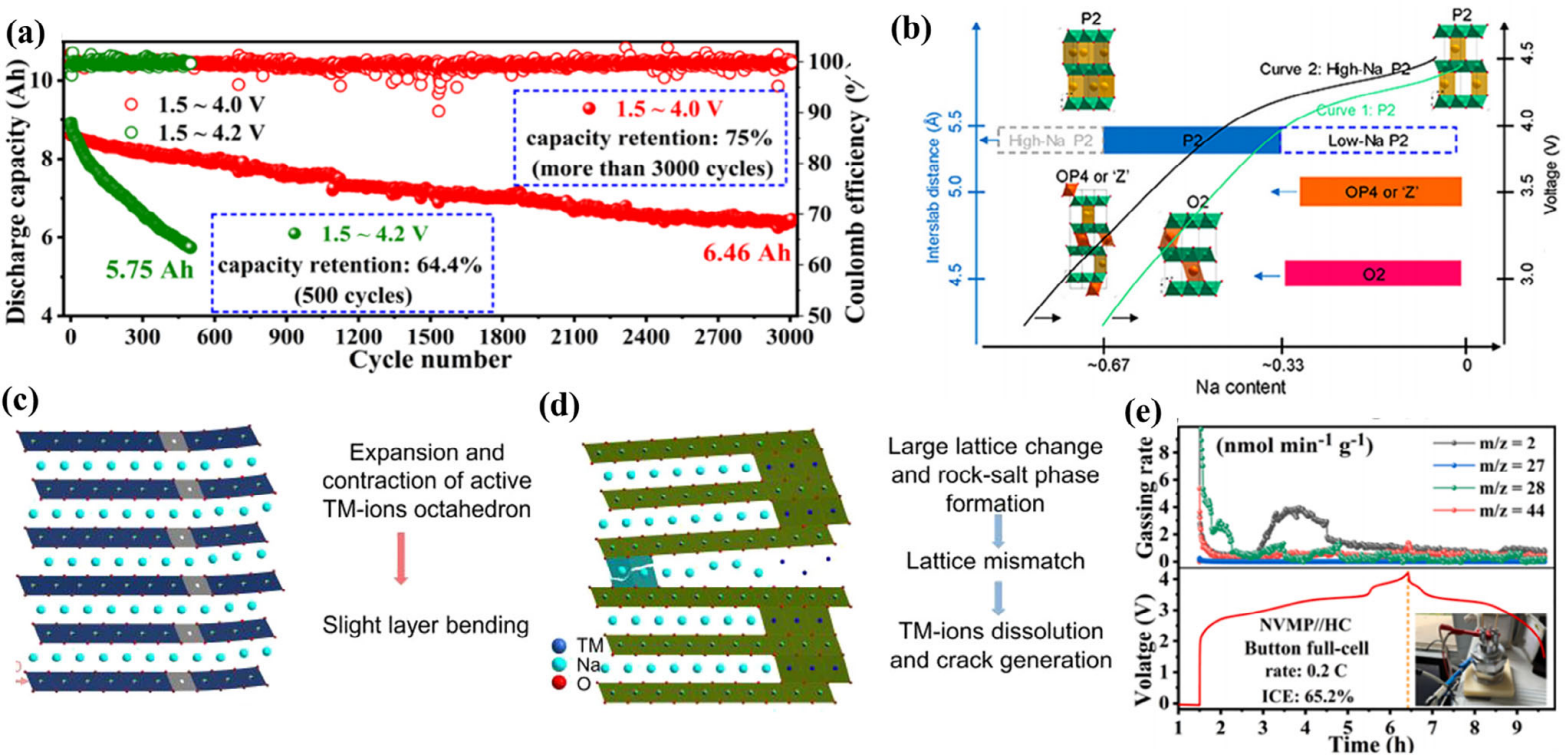
a) Initial charge/discharge cycling performances after cycling tests within the voltage range of 1.5–4.2 V. a) Reproduced with permission.^[^ ^129^ ^]^ Copyright 2024 Elsevier B.V. b) Schematic diagram of irreversible phase transition of P2. Reproduced under terms of the CC‐BY license.^[^ ^128^ ^]^ Copyright 2024, The Authors, published by John Wiley and Sons. c) Schematic illustration of the structural evolution of active TM‐ions octahedron and d) TM‐ions dissolution. Reproduced with permission.^[^ ^153^ ^]^ Copyright 2022 American Chemical Society. e) DEMS mass signals of NVMP//HC full‐cell. Reproduced with permission.^[^ ^129^ ^]^ Copyright 2024 Elsevier B.V.

The bulk phase degradation of cathode materials under high voltage mainly includes irreversible phase transformations, oxygen evolution, cation migration, and transition metal ion leaching, which accelerate capacity fade and impedance growth. The charging cutoff voltage was found to have a significant effect on the cycling performance of polyanionic materials, Tang and others investigated the failure mechanism of pouch cells prepared with Na_3.5_V_1.5_Mn_0.5_(PO_4_)_3_ (NVMP) polyanionic cathode and HC anode.^[^ ^129^ ^]^ As shown in **Figure** **13**a, for NVMP pouch batteries with >3000 cycles in the 1.5–4 V range, the main factors contributing to capacity degradation are “sodium deficiency” and the irreversible inactivation of manganese ions. However, the stable structure of Na_3_V_2_(PO_4_)_3_ and its good Na^+^ diffusion channels prevent the “sodium‐deficient” state from destroying the spent cathode's crystal structure or significantly increasing cathode impedance and worsening Na^+^ diffusion dynamics. Thus, “pre‐sodiumization” and “sodium replenishment” are crucial for extending SIBs’ cycle life and recycling spent SIBs.^[^ ^138^ ^]^ More researchers have reported the high‐voltage bulk phase failure mechanism of layered oxide‐based cathode materials compared to polyanion‐based cathode materials. For P2‐type layered oxides, an irreversible P2‐O2 phase transition (Figure 13b) occurs in the high‐voltage region (>4 V), inducing a lattice volume expansion of ≈23%, leading to structural damage, particle fracture, and microcracking.^[^ ^139^ ^]^ This volume change originates from the weakening of the shielding effect of Na^+^ at high voltages, which leads to an increase in the repulsive forces between the TM layers, which may cause dramatic contraction and expansion of the cathode material structure due to the sliding of the TM layers (Figure 13c). After several electrochemical processes, lattice distortions and cracks are generated, leading to a decrease in electrochemical properties.^[^ ^128^ ^]^ Moreover, this phase transition leads to microcracks and crystal plane bending within the material, which not only hinders Na^+^ transport but also increases the internal stresses, further accelerating structural degradation. As a result, it gets harder to remove Na^+^ during high‐voltage cycling, which causes the capacity to decline quickly.^[^ ^140^, ^141^ ^]^

In addition, TM ions may migrate into the neighboring sodium layer during charging, and this cation migration is particularly common in some O3‐type layered cathode materials.^[^ ^142^ ^]^ Wang and co‐workers discovered that O3‐type layered oxides undergo complex phase transitions such as O3→O“3→P3→P”3→O1.^[^ ^143^ ^]^ They found the O1 phase in NM materials at high voltage, and this phase transition leads to a sharp contraction of the lattice spacing, which further triggers cracking and phase separation. In the high‐voltage region, the reactive metal cations are oxidized to higher valence states, forming weaker TM─O bonds. At the same time, the Na^+^ in the sodium layer is depleted, providing a suitable pathway for the migration of TMs.^[^ ^144^ ^]^ These factors accelerate the migration of TM ions, leading to voltage lag in the cathode material, irreversible anion redox reactions, and poor cycling stability.^[^ ^145^ ^]^ As shown in Figure 13d demonstrates a schematic of the dissolution of TM ions in a layered oxide cathode material.

#### 4.2.2 Interface Degradation

Cathode materials under high voltage also experience challenges of interfacial degradation, which includes problems such as grain boundary cracking, reactive oxygen deficiency, and surface degradation. The drastic phase transition under high voltage can trigger anisotropic internal stresses and lattice distortions leading to crystal cracking. This cracking will continue to extend into the crystal interior, leading to the pulverization of the cathode material. This also destroys the integrity of the CEI film, and cracks are created to provide new contact surfaces for the electrolyte, leading to continued electrolyte depletion and continuous growth of the CEI film. This condition increases the thickness of the CEI film, which in turn increases the interfacial resistance.^[^ ^146^ ^]^ In addition, the side reactions on the cathode surface become more severe under high voltage, including gas emission, transition metal dissolution, and electrolyte decomposition processes. This process further promotes the breakdown of the electrolyte, damaging the crystal arrangement and compromising the integrity of the CEI layer.^[^ ^147^ ^]^ For many layered oxide cathodes, oxidation of the lattice oxygen (O_2_^−^→ O^n−^) is an unavoidable event at elevated voltages.^[^ ^148^ ^]^ The resulting O^n−^ species can easily oxidize to O_2_, causing irreversible oxygen depletion and TM migration, which leads to vacancy formation and further accelerates the oxidation of lattice oxygen, disrupting the lattice framework.^[^ ^149^ ^]^ Additionally, the released O_2_ may interact with the electrolyte, initiating harmful side reactions that contribute to electrolyte degradation and CEI membrane failure, worsening irreversible capacity loss.^[^ ^150^ ^]^ Furthermore, the interfacial materials are not sufficiently stable at high voltages and may continue to decompose, react aggressively with impurities present on the cathode surface, or dissolve in the electrolyte. These processes increase the vertical and lateral inconsistency of the components within the CEI, potentially resulting in localized thermal aggregation and structural breakdown.^[^ ^39^ ^]^

The decomposition of electrolytes under high voltage greatly affects the stability and safety of secondary batteries. For example, ether‐based electrolytes oxidize slightly at low voltages but undergo violent oxidative decomposition at high voltages, especially at voltages above 4 V versus Na/Na^+^, forming an unstable CEI with continuum electrolyte decomposition, surface reconstruction, and capacity decline. At the same time, the low flash point and high flammability of ethers increase the thermal risk, especially at high temperatures, which can cause safety issues and battery degradation.^[^ ^117^ ^]^ Besides, ester‐based electrolytes can maintain strong electrochemical stability without severe oxidative decomposition at low operating voltages (<3.5 V).^[^ ^39^ ^]^ However, solvents and sodium salts in carbonate‐based electrolytes also undergo oxidative decomposition at high temperatures and high voltages, leading to gas production in SIBs during cycling, which causes bulging and deformation of the battery and leads to poor lamination of the pole pieces prior to the electrodes, causing the capacity degradation of the battery.^[^ ^118^ ^]^ It was found that decomposition of the NaPF_6_ electrolyte occurs in the voltage interval of 4–4.2 V, resulting in poor cycle life and swelling gas production. As shown in Figure 13e, a differential electrochemical mass spectrometer (DEMS) analysis of the initial charging of the NVMP//HC full cell. Significant gas evolution H_2_ (m/z = 2, possibly originating from the reduction of R‐OH_2_ or decomposition of H_2_O) and C_2_H_4_ (m/z = 28, possibly originating from the reduction of EC) were observed prior to charging to 4 V. These gases will be completely removed after the cell is formed. However, the slight evolution of CO_2_ at 4.2 V (m/z = 44, originating from the oxidation of EC) suggests that the failure mechanism of NVMP pouch batteries in the range of 1.5–4.2 V is mainly due to the slight oxidation of the electrolyte at the end of each charge, which leads to macroscopic “swelling” of the batteries after an accumulation of 500 cycles.^[^ ^138^ ^]^

#### 4.2.3 Battery Accessories

In addition to the main failure mechanisms of cathode materials described above, the performance of battery accessories such as binders, diaphragms, and gaskets under high voltage also affects the electrochemical performance of the battery. Reasonable selection and configuration of accessories can improve battery safety, cycle stability, and energy density, while inappropriate selection may accelerate the failure of cathode materials and reduce battery performance. Among them, the binder serves as a connecting bridge between the electrode material and the conductive agent, and the collector, and its stability directly affects the stability of the electrode interface. The electrochemical stability of the binder itself is crucial to the performance of high‐voltage batteries. If the binder undergoes chemical decomposition or physical destruction under high voltage, it will lead to poor contact at the interface between the cathode material and the electrolyte, accelerating the battery performance degradation. For example, polar groups in the binder may react with sodium salts or solvents in the electrolyte, leading to changes in the composition and structure of the CEI layer, which in turn affects the battery performance.^[^ ^151^ ^]^ Moreover, the thermal stability of the binder has an important effect on the battery performance under high‐temperature conditions. If the binder is prone to decomposition at high temperatures, it will accelerate the failure of the cathode material and affect the thermal stability and safety of the battery.^[^ ^152^ ^]^ There has been a scarcity of studies on the failure mechanisms of diaphragms and spacers under high‐voltage conditions. However, investigating and optimizing these components is a crucial aspect of advancing high‐voltage SIBs. For example, the change of void structure of diaphragm under high voltage will affect the ion transport and electrolyte adsorption, the change of material volume under high voltage will lead to the rupture of diaphragm and can't work properly, and the thermally unstable gaskets under high voltage may decompose under high temperature, etc., which also affect the safety and stability of the battery.

### 4.3 Feasibility of Commercializing These Materials

The inexpensive cost of raw materials is a major benefit of polyanionic cathode materials like NaFeSO_4_.^[^ ^154^ ^]^ The essential elements of NaFeSO_4_, iron, and sulfates, are found in large quantities in nature and are reasonably priced. However, high‐temperature treatment is frequently needed for their synthesis, which raises production costs and energy consumption.^[^ ^155^ ^]^ These materials are frequently produced using the energy‐intensive but proven solid‐state reaction process, which works well for large‐scale manufacturing.^[^ ^156^ ^]^ Although liquid‐phase techniques can provide more homogeneous and controllable nanoscale porous particles, they may also call for more sophisticated equipment and chemical reagents. Large‐scale production requires high‐temperature furnaces and ball mills, which are well‐established and reasonably priced on the market. Crucially, because polyanionic materials are less susceptible to air and moisture, stricter environmental control during production is not required, which helps to cut costs.

Transition metals including nickel, manganese, and cobalt are commonly used in layered transition metal oxides, which are renowned for their high energy density and suitability for high‐performance batteries. The comparatively high cost of certain metals—particularly cobalt and nickel—raises the price of raw materials.^[^ ^157^ ^]^ Furthermore, heterometal doping and surface coating are frequently needed for performance enhancement, which raises expenses. Large‐scale production can be achieved with the widely utilized high‐temperature solid‐state reaction process, but it necessitates rigorous reaction condition control to guarantee product homogeneity and performance consistency.^[^ ^6^ ^]^

Prussian blue analogs, including NaMnFe (CN)_6_, employ cheap manganese and iron. Although cyanide ligands are harmful, they are reasonably inexpensive. Typically, synthesis uses liquid‐phase techniques like hydrothermal or coprecipitation, which are straightforward and appropriate for large‐scale production but necessitate rigorous reaction condition control.^[^ ^158^ ^]^

In conclusion, polyanionic cathode materials and Prussian blue analogs exhibit significant commercial promise for large‐scale energy storage devices when cost and scalability are taken into account. High‐performance battery applications are better suited for layered transition metal oxides. To speed up the commercialization of these materials, future research should concentrate on lowering costs, improving material stability and electrochemical performance, and streamlining synthesis procedures.

## 5 Conclusion and Perspective

This review explores the advances and challenges associated with high‐voltage cathode materials for SIBs, focusing on their role in improving energy density and performance. We begin by analyzing the key challenges encountered when increasing the upper voltage cutoff, including phase transitions, metal cation migration, and dissolution, loss of reactive oxygen species, and electrolyte interface reactions. The review also discusses various strategies to enhance the high‐voltage stability of cathode materials, such as elemental doping, surface coatings, adjusted synthesis methods, and electrolyte optimization. By lowering the diffusion barrier for Na^+^, elemental doping like Mg, Al, and Ti, improving ion mobility and boosting battery capacity. But it can also change electrical conductivity or lead to structural instability. Moreover, overdoping may lead to phase segregation and unsatisfied electrochemical performance. By adjusting doping concentration and selecting dopants based on theoretical predictions and experimental confirmation, conductivity, and stability may be matched. Computational modeling can help identify the best element combinations for enhanced performance. Surface modifications enhance electrical conductivity, improve chemical stability, and reduce side reactions with the electrolyte. However, while surface coatings generally improve stability, they may also reduce the material's tap density, potentially sacrificing some energy density. Additionally, certain coatings may degrade over prolonged cycling, leading to a loss of effectiveness. The development of self‐healing or adaptable coatings that react dynamically to electrochemical conditions may enhance long‐term stability. Refined synthesis methods enable precise control over the material's microstructure and morphology, optimizing particle size for better Na^+^ transport and electrochemical performance. Using precise synthesis techniques to control microstructure and morphology may be costly and challenging. Moreover, the scalability of advanced synthesis techniques (such sol–gel, hydrothermal, or solid‐state) may limit their industrial use. While maintaining desirable structural features, commercial viability can be increased by improving synthesis repeatability and looking into scalable, reasonably priced methods such as spray pyrolysis or template‐assisted synthesis.

**Figure 14:**
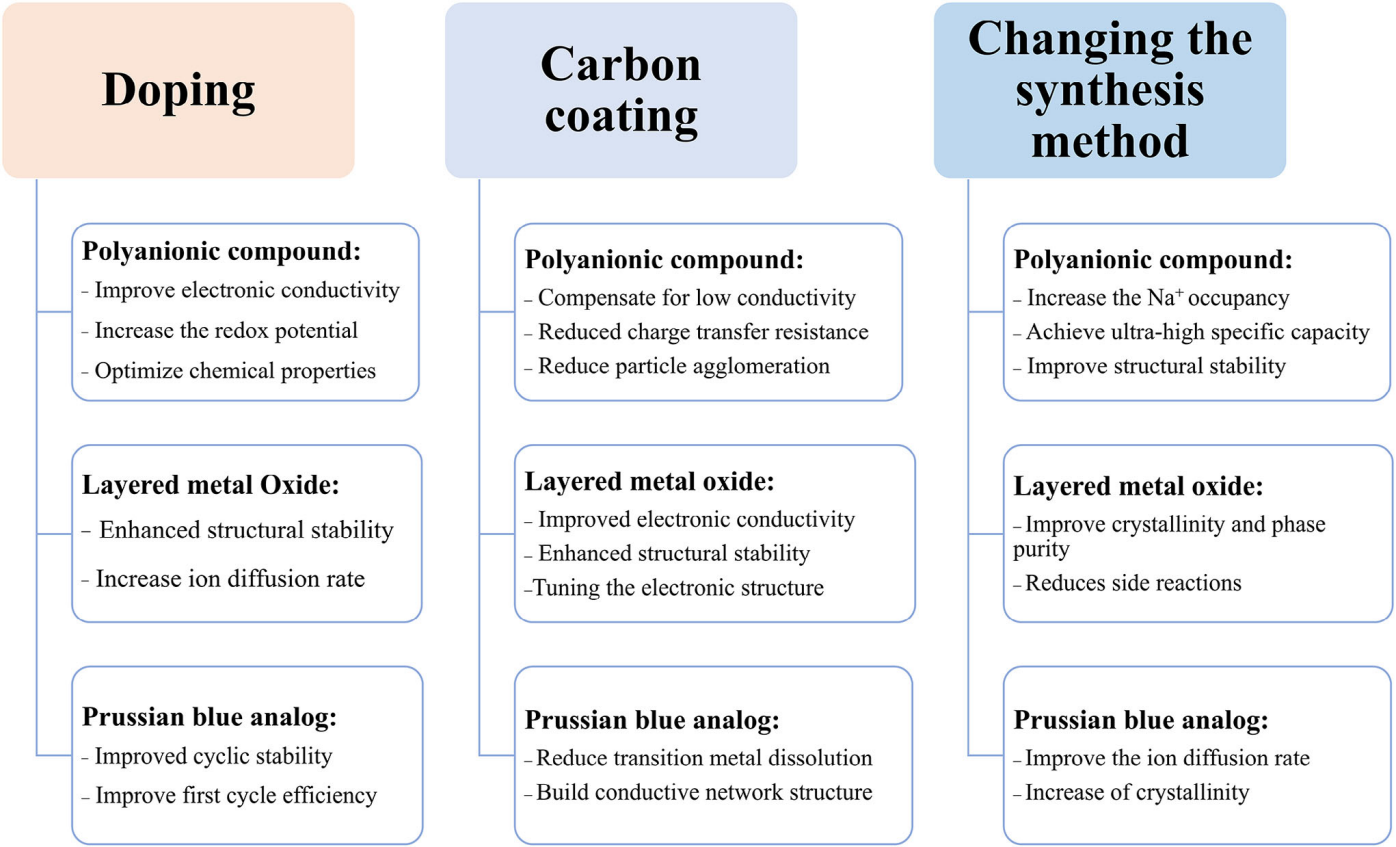
A brief discussion on the three main structures of SIB cathode materials under the same optimization strategy.

As shown in **Figure** **14**, the optimization strategies for the three main structures of SIB cathode materials have led to advancements in high‐voltage cathode development. Enhancing structural stability, commercial viability, and electrochemical performance are the goals of these tactics. Surface coatings, cation doping, and anion substitution have all helped layered transition metal oxides by reducing structural deterioration and enhancing cycle stability. However, challenges persist in optimizing electrolyte compatibility and reducing reliance on expensive transition metals such as Co and Ni. Polyanionic compounds offer excellent structural stability and high operating voltages due to strong covalent bonding. However, their low electronic conductivity and energy‐intensive synthesis processes hinder practical applications. Current research focuses on hybrid synthesis methods, carbon coatings, and partial anionic substitution to improve conductivity while reducing production costs. PBAs have emerged as reliable, open‐framework, and cost‐effective alternatives. However, their full potential is limited by low electronic conductivity and challenges in water content regulation. Further advancements in surface modification, controlled synthesis, and electrolyte engineering will be essential to improve their electrochemical performance.

Moreover, electrolyte optimization is essential to improve conductivity, stability, and prevent electrolyte decomposition, ensuring the protection of the electrode material. The development and matching of the electrolyte system increases the conductivity and stability of the cathode material, and a well‐designed CEI can effectively prevent the decomposition of the electrolyte, protect the electrode material, and reduce the capacity decay; The use of additives can improve the wettability of the electrolyte, promote the contact between the electrolyte and the electrode material, and enhance the overall performance of the battery; Designing (e.g., electrode thickness, diaphragm material, electrode material and electrolyte, etc.) a suitable full cell can improve the energy density of the battery and ensure its reliability in practical applications.

Although significant progress has been made in recent years in improving the high‐voltage stability of cathode materials, there are still serious challenges in further improving the cycling stability and multiplication performance.

1. Future research must thoroughly investigate the electrochemical behavior of cathode materials in high‐voltage settings, including crucial processes like ion diffusion, electron transfer, and phase transitions, in order to address the deterioration of these materials under high voltage. Through these studies, we may gain a deeper understanding of the intrinsic relationship between cathode material performance and structure at high voltage. This comprehensive knowledge is crucial for advancing the creation of new cathode materials, particularly those with high output voltage and high specific capacity, which hold great promise for energy storage applications in the future.
2. Electrolyte stability is essential for battery performance in high‐voltage settings. In order to reduce electrolyte breakdown and gas production and to increase battery cycle stability, it is imperative that electrolytes compatible with high‐voltage cathode materials be developed. We offer a comprehensive understanding of the physicochemical processes at the electrolyte/electrode interface and uncover the microscopic mechanisms of the interfacial reactions by combining multi‐scale simulation and experimental validation. This will theoretically support the optimization of battery design. In order to satisfy the need for high‐performance batteries in the next energy market, we also study and create artificial CEI layers, such as ion‐conductive inorganic membranes, polymer membranes, and their composites.
3. Researchers systematically investigate the thermal stability and electrochemical behaviors of high‐voltage cathode materials at various temperatures in order to better understand the failure mechanisms of these materials under extreme temperature conditions (such as very low and very high temperatures). They must also develop strategies to increase the safety of high‐voltage cathode materials and strike a balance between ensuring safety and increasing energy density. Researchers must create effective cooling methods and materials with greater thermal stability in the interim to solve the thermal runaway issue. They must also construct sophisticated battery management systems to further stop thermal runaway incidents.
4. More in situ and operando techniques such as in situ atomic force microscopy (AFM) and cryo‐electron microscopy, operando TEM, and X‐ray tomography (XCT) are needed to fully understand the relationship between the structure and properties of materials. Synchrotron XAS/XRD and nuclear magnetic resonance (NMR) are needed to study the local chemical environment and dynamic information of specific elements in the material; and thermal analytical techniques such as differential scanning calorimetry (DSC) and thermogravimetric analysis (TGA) are needed to measure the material's thermal properties.
5. In accelerating the development and optimization of new materials, theoretical models and computational methods, such as machine learning algorithms and artificial intelligence, should be combined to simplify the complex reaction process, reduce the cost of trial and error, and predict the crystalline structure, electrochemical properties, and stability of new materials. Promote the development of laboratory research results toward scale‐up and commercialization to achieve high‐performance, high‐stability, and low‐cost SIB technology.

Research on high‐voltage cathode materials is not only important for improving battery performance, but also has broad application prospects in promoting energy transition and achieving carbon neutrality, such as providing a longer range for electric vehicles; providing high energy density and high‐reliability power solutions in the military or aerospace fields; and serving as a temporary power source in emergency situations. With further research and technological advances, it is expected that these materials will play an increasingly important role in future energy systems.

## Conflict of Interest

The authors declare no conflict of interest.
